# Deviations from classical droplet evaporation theory

**DOI:** 10.1098/rspa.2021.0078

**Published:** 2021-07

**Authors:** Joshua Finneran, Colin P. Garner, François Nadal

**Affiliations:** Wolfson School of Mechanical Electrical and Manufacturing Engineering, Loughborough University, Loughborough LE11 3TU, UK

**Keywords:** droplet vaporization, fully transient, unsteady, quasi-steady, Stefan flow, moving boundary

## Abstract

In this article, we show that significant deviations from the classical quasi-steady models of droplet evaporation can arise solely due to transient effects in the gas phase. The problem of fully transient evaporation of a single droplet in an infinite atmosphere is solved in a generalized, dimensionless framework with explicitly stated assumptions. The differences between the classical quasi-steady and fully transient models are quantified for a wide range of the 10-dimensional input domain and a robust predictive tool to rapidly quantify this difference is reported. In extreme cases, the classical quasi-steady model can overpredict the droplet lifetime by 80%. This overprediction increases when the energy required to bring the droplet into equilibrium with its environment becomes small compared with the energy required to cool the space around the droplet and therefore establish the quasi-steady temperature field. In the general case, it is shown that two transient regimes emerge when a droplet is suddenly immersed into an atmosphere. Initially, the droplet vaporizes faster than classical models predict since the surrounding gas takes time to cool and to saturate with vapour. Towards the end of its life, the droplet vaporizes slower than expected since the region of cold vapour established in the early stages of evaporation remains and insulates the droplet.

## 1. Introduction

Since Maxwell’s seminal work [1], continued commitment to understand evaporation processes has fostered a rich scientific literature on topics as diverse as spray cooling, combustion, climate science, medical treatments, cosmetics and manufacturing processes [2–6]. The most elementary droplet evaporation configuration, of a single, spherical, pure droplet in a quiescent, infinite, isotropic, gaseous medium is a foundational problem, the understanding of which is fundamental to addressing more complex systems (e.g. non-spherical [7], moving droplets [8–11], multi-component droplets [11–14] and droplet clusters [15–17]). This article addresses this important fundamental problem and the scope of the following discussion is confined to studies that present key developments in the understanding of this specific problem. The earliest solution, provided by Maxwell [1] and later by Langmuir [18], considers purely diffusive transports in the surrounding gas phase and requires assuming steadiness of the liquid droplet and the surrounding gas. This reduces the problem to a steady heat and mass diffusion problem and the mass equation solves to give the evaporative mass flux $j_{\text{ev}}$ as

1.1 $$j_{\text{ev}}=\frac{\Gamma }{a}(\omega_{s}-\omega_{\infty }),$$

where a is the droplet radius, $\omega_{s}$ and $\omega_{\infty }$ are the vapour mass fraction at the surface and far from the droplet, respectively. The mass diffusivity Γ is assumed constant and is the product of gas density ρ and molecular diffusivity D. Fuchs [19] extended this solution to account for the net convective velocity in the gas (Stefan flow), which results in

1.2 $$j_{\text{ev}}=\frac{\Gamma }{a}\ln\left(1+\frac{\omega_{s}-\omega_{\infty }}{1-\omega_{s}}\right).$$

Integration of equations (1.1) and (1.2) results in the classical $d^{2}$-law (i.e. the droplet diameter-squared reduces linearly in time), but with differing gradients. It should be a feature of all more advanced solutions that they recover or approach solutions obtained with a reduced number of assumptions in a limiting case, e.g. equation (1.2) approaches equation (1.1) in the limit $\omega_{s}\to 0$.

The description in equations (1.1) and (1.2) is incomplete since the surface concentration $\omega_{s}$ is generally unknown and is determined from the surface temperature $T_{s}$, which in turn depends on solution to the energy equation [20]. The gas temperature can vary substantially between the droplet surface and the ambient. The steady coupled heat and mass transfer problem has been solved for a non-isothermal gas phase [21,22]. However, it is important to note that obtaining equation (1.2) does not require the isothermal gas assumption, but only requires that Γ=ρD is constant [20] (see also electronic supplementary material, S3).

A notable feature of equation (1.2) is that this solution breaks down for small droplets, since $j_{\text{ev}}\to \infty$ for a→0. This problem was also addressed by Fuchs [19], and later by Bradley *et al.* [23], by introducing a correction based on the mean free path length in the gas phase $l_{\text{coll}}$. For droplets at the scale of $l_{\text{coll}}$, the fluid can no longer be treated as a continuum and molecular dynamics becomes significant. The Knudsen number $\text{Kn}=l_{\text{coll}}/a$ quantifies the significance of kinetic effects. For large Knudsen numbers (Kn∼1), experiments and molecular dynamics simulations have shown there is a temperature *jump* at the liquid–vapour interface that is inversely proportional to the vapour density [24]. This temperature jump refers to a rapid drop in temperature from the vapour to the liquid on the scale of individual molecules, over the so-called *Knudsen layer*. Rana *et al.* [25] implemented a temperature jump boundary condition in modelling small droplets ($a∼10^{-8}\;\text{m}$) and showed this leads to a departure from the $d^{2}$-law toward a *d*-law. At the opposite scale, for large droplets (approx. $10^{-4}$ m), deviations from the $d^{2}$-law have been demonstrated due to radiative heat transfer at high ambient temperatures [26].

An often-encountered practical scenario is where a droplet is delivered into a higher temperature gas. The liquid is generally colder than its steady *wet-bulb* temperature so the droplet heats up while evaporating. Classical models accounting for heating, or cooling in some cases, can assume an infinite liquid conductivity so the liquid temperature is homogeneous, or can consider a finite conductivity when accounting for temperature gradients within the droplet [27]. Effective conductivity models can be used to account for internal circulation within the droplet [28]. These models show that, while the droplet is heating, there is a deviation from the $d^{2}$-law, but once the wet-bulb temperature is reached, $d^{2}$-law behaviour emerges. Talbot *et al.* [29] provided dimensionless criteria for determining the significance of the liquid phase transients (droplet heating) corresponding to the response of the droplet surface and bulk internal temperature.

The preceding models that result in $d^{2}$-law behaviour once the steady droplet temperature is reached are based on the classical theory assumption that the gas is *quasi-steady* (QS). Models that remove this assumption are referred to as *fully transient*. The implications of removing the QS assumption are the major topic of this article. When a liquid droplet is suddenly exposed to an undisturbed atmosphere, there is initially a large vapour concentration gradient at the droplet surface leading to rapid mass diffusion and so evaporation. As time advances, a layer of vapour builds up around the droplet that reduces with distance and this attenuates the evaporation rate. Simultaneously, there is an analogous effect with temperature since the gas surrounding the droplet must be cooled. The simultaneous heat and mass transfer processes in the gas phase are important, complementary processes in droplet evaporation problems. The classical QS models, therefore, neglect these transient processes in the gas phase and assume that the steady vapour concentration and temperature fields are established instantaneously.

The classical justification for the QS assumption originates from comparing time scales of evaporation with the mass and thermal diffusion time of the gas phase [2]. The ratio of mass diffusion time to evaporation time and thermal diffusion time to evaporation time suggests that the QS assumption should be valid for $\rho_{\infty }/\rho_{l}\ll 1$ and $\rho_{\infty }/(\text{Le}\rho_{l})\ll 1$, respectively, where $\rho_{\infty }$ is the ambient density, $\rho_{l}$ is the liquid density and Le is the Lewis number [22]. This justification predicts its own demise as the density ratio increases, for example, at high pressure. Also, there are logical inconsistencies with the QS assumption such as the infinite vapour and temperature boundary layer that requires infinite mass and energy to establish (further details provided in electronic supplementary material, S4).

In terms of fully transient models, Hubbard *et al.* [30] modelled evaporation of octane droplets in air at pressures up to 10 atm and concluded that gas phase transient effects were negligible and independent of droplet size. Zhu *et al.* [31] considered n-heptane evaporation in nitrogen for a wide range of gas temperatures and pressures. They showed that the difference between the QS and fully transient models increased with pressure and ambient temperature. Azimi *et al.* [32] modelled fully transient evaporation of various hydrocarbons at atmospheric pressure and determined that the QS model overpredicts the evaporation time and the overprediction increases for increased ambient temperature, for heavier fuels and for increased droplet sizes. However, the number of cases was limited to three fuels at two temperatures. Tonini & Cossali [33] modelled evaporation of various fluids under ambient conditions of $1\;\text{bar}/500^{\circ }\text{C}$. They found that the effect of a moving droplet surface can cause a 20% difference in the time to reach 95% of initial droplet size between the QS and fully transient models.

In the previously reported literature on the present problem, a variety of different combinations of assumptions are used, some of which are not always clearly stated, which makes generalized conclusions challenging to draw and crucially limits the fundamental understanding that can be gained. Moreover, the previous investigations tend to use specific fluids, generally heavy hydrocarbons in air, which makes the findings application specific. The present article fundamentally addresses this problem by considering a generalized fluid system governed by dimensionless numbers and with all assumptions clearly stated within the text as well as being listed in appendix A. To the present authors’ knowledge, the bounds under which the QS assumption is valid have not previously been robustly defined. The closest finding in this regard is that the error caused by the QS assumption is of the order of the square root of the gas/liquid density ratio ${\left(\rho_{\infty }/\rho_{l}\right)}^{1/2}$ [34]. As new technologies emerge, with different operating conditions and working fluids, the QS assumption cannot necessarily be taken for granted. Therefore, it would be valuable to have a clear, fundamental methodology for determining its validity. The study presented in this article fulfils this need by considering many generalized cases (approx. 1000), compared with the few in previous studies. Additionally, this study contributes towards understanding the effects of a moving droplet surface and evaporation at elevated, near-critical pressures, which were identified by Shazin [6] as unsolved problems in evaporation. Other features that separate the present work from previous studies include advancing the solution until the droplet has reached 1% of its original diameter that reveals new dynamic effects towards the end of droplet life. Gas phase transient effects are studied in isolation of liquid phase transients (droplet heating and cooling) by careful selection of initial conditions. This reveals that significant deviation from the classical theory can occur solely due to gas phase transients. Additionally, the effects of initial conditions are analysed showing that the gas phase transient effects that manifest are independent of initial conditions.

This article is structured as follows. Firstly, the governing equations and boundary conditions of the problem are defined and made dimensionless. Past solutions are recovered and it is ensured that the present model approaches the QS solution in the limiting case. In terms of results, the characteristics of fully transient evaporation are described and physically explained, followed by presenting a predictive tool for quantifying errors caused by the QS assumption. Finally, the findings are applied to a range of fluids evaporating in air. The dimensionless approach and large number of cases considered allows general conclusions to be made that increase the fundamental understanding of droplet evaporation physics.

## 2. Problem definition

**Figure 1.**
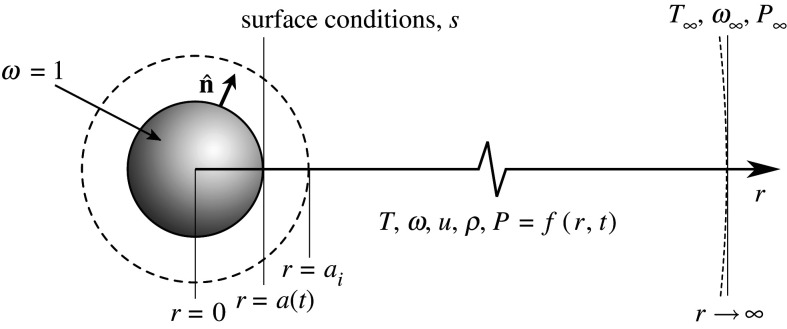
Illustration of the general droplet evaporation problem. The droplet is pure (mass fraction ω=1) and begins with radius $a_{i}$. The droplet radius a varies in time and the property fields vary in space and time.

The problem being addressed here is the evaporation of a single, spherical, pure (species A) droplet in a stagnant, infinite, isotropic gaseous medium (mixture of species A and B) as illustrated in figure 1. For a given initial state, the task is to predict the droplet size history and the temperature, mass fraction, velocity and pressure field history. This section introduces the governing equations and boundary conditions in their full, unsimplified form, invoking as few assumptions as is practical. The system is then made dimensionless, which provides criterion for justifying the assumptions used.

### (a) Governing equations and boundary conditions

The governing mass, species, momentum, energy and state equations are now presented. The total mass equation is

2.1 $$\frac{\partial \rho }{\partial t}+\nabla \cdot (\rho u)=0,$$

where ρ is density, u is the velocity vector and t is time. The continuity equation for species A is

2.2 $$\rho \frac{\text{D}\omega }{\text{D}t}=\nabla \cdot (\Gamma \;\nabla \omega ),$$

where D/Dt is the material derivative, ω is the mass fraction of the evaporating species (species A) and the mass diffusivity Γ is the product of molecular diffusivity and density Γ=ρD. Note that the mass fraction of species B is 1−ω. The mass diffusivity is used throughout this analysis rather than the molecular diffusivity due to its lower sensitivity to temperature (from kinetic theory of gases, $\Gamma ∼T^{1/2}$, whereas $D∼T^{3/2}$ [35]). The momentum equation is

2.3 $$\rho \frac{\text{D}u}{\text{D}t}=-\nabla P+\nabla \cdot \sigma +\rho g,$$

where P is the pressure, σ is the viscous stress tensor and g is the gravitational acceleration vector. The energy equation in temperature form is given by

2.4 $$\rho c_{p}\frac{\text{D}T}{\text{D}t}-\frac{\text{D}P}{\text{D}t}=\nabla \cdot (\lambda \;\nabla T)+\Delta c_{p}\Gamma \;\nabla \omega \cdot \nabla T+\sigma :\nabla u,$$

where $c_{p}$ is the local constant pressure specific heat, λ is the thermal conductivity, $\Delta c_{p}$ is the difference in specific heat between the evaporating (superscript A) and ambient (superscript B) species ($\Delta c_{p}=c_{p}^{A}-c_{p}^{B}$). The term $\Delta c_{p}\Gamma \;\nabla \omega \cdot \nabla T$ is energy transport due to mass diffusion and σ:∇u is viscous dissipation. It is the authors’ opinion that derivation of the energy equation in this form is not trivial and is not provided in detail elsewhere, so its derivation has been included in the electronic supplementary material, S1. At this stage, the gas phase system of equations (2.1)–(2.4) are applicable to any ideal binary fluid mixture. These equations are therefore applicable to the liquid phase by simply replacing the fluid properties with those of the liquid, before significant simplification later. The system of equations (2.1)–(2.4) is closed with the ideal gas equation of state, written as P=ρRT, where R is the specific gas constant.

Fixed conditions are assumed at a far distance from the droplet, therefore $T\to T_{\infty }$, $\omega \to \omega_{\infty }$ and $P\to P_{\infty }$ for r→∞. Through the state equation, this also defines the far-field density, $\rho_{\infty }$.

Assuming that the ambient species are insoluble in the liquid droplet, the mass flux of the ambient species at the surface relative to the moving droplet surface $j_{\text{rel},s}^{B}$ is zero, i.e. $j_{\text{rel},s}^{B}\cdot \hat{\text{n}}=0$, where $j_{\text{rel}}^{B}=\rho (1-\omega )u_{\text{rel}}+\Gamma \nabla \omega$, $\hat{\text{n}}$ is a unit vector normal the the droplet surface, subscript s indicates at the droplet surface and $u_{\text{rel}}$ is the velocity relative to the droplet surface. The evaporative mass flux $j_{\text{ev}}$ is defined as the mass crossing the droplet surface boundary, hence $j_{\text{ev}}\equiv j_{\text{rel},s}^{A}$. Therefore, $j_{\text{ev}}\cdot \hat{\text{n}}=\rho_{s}u_{\text{rel},s}\cdot \hat{\text{n}}$ and by combining these expressions we obtain the evaporative mass flux in terms of the scalar mass fraction field as

2.5 $$j_{\text{ev}}\cdot \hat{\text{n}}=-\frac{\Gamma }{1-\omega_{s}}{\left[\nabla \omega \right]}_{s}\cdot \hat{\text{n}}.$$

The velocity of the droplet surface (da/dt), where a is the vector position of the droplet surface ($a=a\;\hat{\text{n}}$ for a spherical droplet), can be determined from the evaporative flux through a mass balance by

2.6 $$\frac{\text{d}a}{\text{d}t}=-\frac{j_{\text{ev}}}{\rho_{l}},$$

where $\rho_{l}$ is the droplet liquid density. Assuming that the transition zone between the phases is infinitely thin (negligible Knudsen layer), there is a discontinuity in fluid properties at the surface. Neglecting radiative heat transfer, temperature and energy flux continuity at the droplet surface, respectively, results in

2.7 $$T_{s}^{-}=T_{s}^{+}\;\text{and}\;-\lambda_{l}{\left[\nabla T\right]}_{s}^{-}=-\lambda {\left[\nabla T\right]}_{s}^{+}+j_{\text{ev}}L,$$

where L is the latent heat of vaporization and superscripts − and + indicate properties on the liquid side and the gas phase side of the phase boundary, respectively.

Assuming that the droplet surface is locally in phase equilibrium and neglecting surface tension effects, then $P_{s}^{A}=P_{\text{sat}}(T_{s})$, where $P_{s}^{A}$ is the surface partial pressure of A and subscript ‘sat’ indicates saturation conditions. This relationship is obtained from the Clausius–Clapeyron equation, given by

2.8 $$\ln\left(\frac{P_{s}^{A}}{P_{\infty }}\right)=\frac{L}{R^{A}}\left(\frac{1}{T_{\text{BP}}}-\frac{1}{T_{s}}\right),\;\text{where}\;P_{s}^{A}=P_{s}{\left[1+\frac{M^{A}}{M^{B}}\left(\frac{1}{\omega_{s}}-1\right)\right]}^{-1},$$

and $T_{\text{BP}}$ is the boiling temperature evaluated at ambient pressure $P_{\infty }$. This equation gives high accuracy locally about $T_{\text{BP}}$. For most cases in this study, the surface temperature is always very close to $T_{\text{BP}}$ such that the equation is valid (see initial conditions, §2c). The second expression is useful to relate the partial pressure to the mass fraction, where $P_{s}$ is the pressure at the surface and $M^{A}$ and $M^{B}$ are the molecular masses of the evaporating and ambient species, respectively. This expression is true for an ideal gas mixture since the mole fraction is equivalent to the partial pressure fraction (Dalton’s Law).

### (b) Dimensionless governing equations and boundary conditions

To reduce the number of parameters, the governing equations and boundary conditions are now made dimensionless by introducing typical scales of the system. The typical length $L^{⋆}$ is taken to be the initial droplet radius, the typical velocity $u^{⋆}$ is an order of magnitude for the Stefan flow gas velocity at the initial droplet surface, the typical time $t^{⋆}$ is constructed from the previous length and velocity scales and the typical density $\rho^{⋆}$ is taken as the far-field gas density. Therefore, we select $L^{⋆}=a_{i}$, $u^{⋆}=\Gamma_{\infty }/(\rho_{\infty }a_{i})$, $t^{⋆}=\rho_{\infty }a_{i}^{2}/\Gamma_{\infty }$ and $\rho^{⋆}=\rho_{\infty }$.

It is desired to introduce the temperature, mass fraction and pressure fields differences relative to their far-field values, which defines a new system of variables as $\theta =T-T_{\infty }$, $\varphi =\omega -\omega_{\infty }$ and $P=P-P_{\infty }$. The typical pressure scale is taken as $P^{⋆}∼\rho^{⋆}{\left(u^{⋆}\right)}^{2}=\Gamma_{\infty }^{2}/(\rho_{\infty }a_{i}^{2})$. The temperature and mass fraction scales are, respectively, $\theta^{⋆}=T_{\infty }-T_{\text{Q S},s}$ and $\varphi^{⋆}=\omega_{\text{Q S},s}-\omega_{\infty }$, where $T_{\text{Q S},s}$ and $\omega_{\text{Q S},s}$ are the QS temperature and vapour concentration at the droplet surface, respectively. The subscript ‘QS’ indicates values obtained from the QS solution. The constants $T_{\text{Q S},s}$ and $\omega_{\text{Q S},s}$ have a definition that derives from solving the QS system of equations. For this reason, these values are not new inputs, but are derived from the readily available system parameters, as will be demonstrated in §3.

In the most general case, transport properties and specific heats are also field variables. The typical values for transport properties are taken as the far-field values, i.e. $\Gamma^{⋆}=\Gamma_{\infty }$, $\lambda^{⋆}=\lambda_{\infty }$ and $\mu^{⋆}=\mu_{\infty }$. Specific heats are generally evaluated using polynomial fits with temperature. Expanding about the far-field temperature for gas species β (where β can be A or B), one obtains

2.9 $$c_{p}^{\beta }=c_{p,\infty }^{\beta }+C_{p}^{\beta },\;\text{where}\;C_{p}^{\beta }=\sum_{n=1}^{N}A_{n}^{\beta }{\left(T-T_{\infty }\right)}^{n},$$

and the constant $c_{p,\infty }^{\beta }$ is the specific heat of the species β at infinity and $C_{p}^{\beta }$ is the temperature correction component, the coefficients $A_{n}^{\beta }$ are the polynomial coefficients and N is the order of expansion. The typical specific heat value is taken as the far-field value, so $c_{p}^{⋆}=c_{p,\infty }$. The typical viscous shear is taken as $\sigma^{⋆}=\mu_{\infty }u^{⋆}/L^{⋆}$. Finally, the gravitational acceleration magnitude is the constant g.

In the following, dimensionless variables are denoted with a tilde ($\tilde{X}$). Substituting all variables, the full, unsimplified gas phase governing equations (2.1)–(2.4), respectively, become

2.10 $$\begin{array}{rl} & \frac{\partial \tilde{\rho }}{\partial \tilde{t}}+\tilde{\nabla }\cdot (\tilde{\rho }\tilde{u})=0, \end{array}$$

2.11 $$\begin{array}{rl} & \tilde{\rho }\frac{\text{D}\tilde{\varphi }}{\text{D}\tilde{t}}=\tilde{\nabla }\cdot (\tilde{\Gamma }\;\tilde{\nabla }\tilde{\varphi }), \end{array}$$

2.12 $$\begin{array}{rl} & \tilde{\rho }\frac{\text{D}\tilde{u}}{\text{D}\tilde{t}}=-\tilde{\nabla }\tilde{P}+Sc\tilde{\nabla }\cdot \tilde{\sigma }+\frac{1}{\text{Fr}}\tilde{\rho }\tilde{g} \end{array}$$

$$\begin{array}{rl} \text{and}\; & (1+\chi \tilde{\varphi }+\xi^{A}{\tilde{C}}_{p}^{A}+\xi^{B}{\tilde{C}}_{p}^{A})\tilde{\rho }\frac{\text{D}\tilde{\theta }}{\text{D}\tilde{t}}-\text{Ec}\frac{\text{D}\tilde{P}}{\text{D}\tilde{t}} \end{array}$$

2.13 $$\begin{array}{rl} & \;=\text{Le}\tilde{\nabla }\cdot (\tilde{\lambda }\tilde{\nabla }\tilde{\theta })+[\chi +\Psi ({\tilde{C}}_{p}^{A}-{\tilde{C}}_{p}^{B})]\tilde{\Gamma }\tilde{\nabla }\tilde{\varphi }\cdot \tilde{\nabla }\tilde{\theta }+Sc\;\text{Ec}\;\tilde{\sigma }:\tilde{\nabla }\tilde{u}, \end{array}$$

and the state equation becomes

2.14 $$1+{\text{Ma}}^{2}\tilde{P}=\tilde{\rho }(1+\zeta \tilde{\varphi })(1+\kappa \tilde{\theta }),$$

where Sc, Fr, Ec, Le and Ma are the Schmidt, Froude, Eckert, Lewis and Mach number, respectively, and all dimensionless numbers are defined in table 1. Some dimensionless numbers are redundant for defining the system, i.e. they can be derived from other dimensionless numbers but are still useful for conciseness; these are denoted as ‘dependent’ in table 1. Notably, the Spalding heat and mass transfer numbers, $B_{T}$ and $B_{M}$, respectively, are defined as

2.15 $$B_{T}=\frac{c_{p,\infty }^{A}}{L}(T_{\infty }-T_{\text{Q S},s})\;\text{and}\;B_{M}=\frac{\omega_{\text{Q S},s}-\omega_{\infty }}{1-\omega_{\text{Q S},s}}.$$

The dimensionless numbers $B_{T}$ and $B_{M}$ are especially important in the context of droplet evaporation but are not governing numbers and can be derived readily from other dimensionless numbers, as will be shown in §3. Additionally, the variables $\xi^{A}$ and $\xi^{B}$, relevant to the energy equation, are defined for conciseness as $\xi^{A}=\Psi \tilde{\varphi }-\Phi +1$ and $\xi^{B}=\Psi \tilde{\varphi }-\Phi$.

**Table 1.** Definitions of dimensionless numbers. The numbers ζ and χ describe the significance of how dissimilar A and B are in terms of molecular mass and specific heat respectively. The numbers κ and Ψ give the significance of temperature and vapour concentration changes, respectively. (Note: the Jakob number Ja is usually defined using the liquid phase specific heat, but the present definition is more appropriate in this context.)

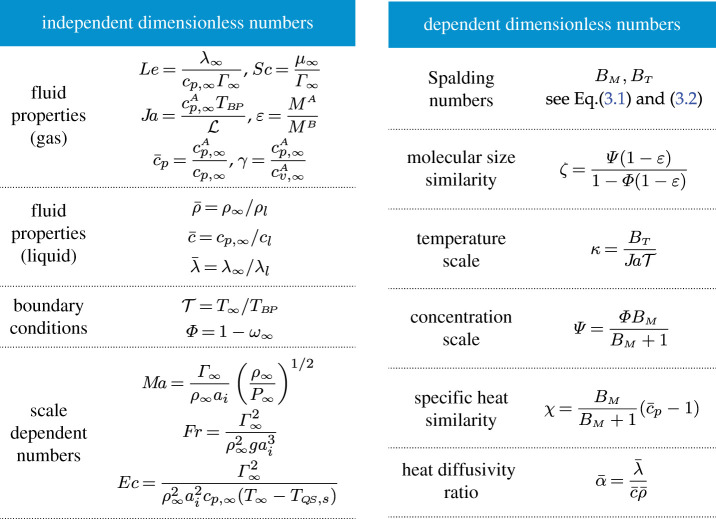

By evaluating all of the dimensionless numbers, the validity of certain assumptions can be assessed. The equations are now reduced by invoking additional assumptions and giving criteria for their validity. Equation (2.9) in dimensionless form is

2.16 $${\tilde{C}}_{p}^{\beta }=\sum_{n=1}^{N}\frac{A_{n}^{\beta }}{c_{p,\infty }}{\left(T_{\infty }-T_{\text{Q S},s}\right)}^{n}{\tilde{\theta }}^{n}.$$

Therefore, evaluating the coefficients $A_{n}^{\beta }{\left(T_{\infty }-T_{\text{Q S},s}\right)}^{n}/c_{p,\infty }$ relative to unity determines the necessary order of expansion. Each gas species will introduce N additional coefficients required to describe the system that will be unique for the gases under consideration. To keep the analysis general, constant specific heats are assumed in the present study, i.e. ${\tilde{C}}_{p}^{\beta }=0$.

For a low-density monatomic gas, transport properties vary with the square root of temperature, i.e. $\tilde{\Gamma }$, $\tilde{\lambda },\tilde{\mu }={\left(T/T_{\infty }\right)}^{1/2}$ or equivalently $\tilde{\Gamma }$, $\tilde{\lambda }$, $\tilde{\mu }={\left(\kappa \tilde{\theta }+1\right)}^{1/2}$. This can be used as a first approximation for more complex molecules. The magnitude of the coefficient κ determines the validity of constant transport properties, which tends to unity for large T. However, as with specific heats, in general, true relationships for transport properties can introduce an arbitrarily large number of additional numbers to fit to experimental data and therefore results obtained with accurate transport property data will be specific to the fluids under consideration. For simplicity and to achieve direct comparison with the classical QS solution, constant transport properties are assumed in the present study (i.e. $\tilde{\Gamma },\tilde{\lambda },\tilde{\mu }=1$). It has been shown that variable properties can have a significant impact on the droplet lifetime [22,30,36] and so for constant properties to be used they must be evaluated at some representative average condition. However, the effects of variable transport properties on the present conclusions are expected to be small because Le is the only relevant number containing transport properties and this has a low sensitivity to the conditions where average properties are evaluated since the temperature dependence of λ and Γ in Le approximately cancel.

Microgravity was assumed and is justified for Fr≫1. This results in spherical symmetry of the problem and therefore $\tilde{\nabla }\equiv \partial /\partial \tilde{r}$. Incompressibility was assumed and is justified for ${\text{Ma}}^{2}\ll 1$. This eliminates density dependence on pressure and decouples the momentum equation from the system of equations (2.10)–(2.14) allowing it to be solved independently for the pressure field if desired. Work terms were neglected, which is justified for Ec≪1. This eliminates the pressure and viscous work terms in the energy equation. The Schmidt number Sc also becomes redundant since the momentum equation is decoupled.

With these assumptions, the system equations (2.10)–(2.14) reduce to

2.17 $$\begin{array}{rl} & \frac{\partial \tilde{\rho }}{\partial \tilde{t}}+\frac{1}{{\tilde{r}}^{2}}\frac{\partial }{\partial \tilde{r}}({\tilde{r}}^{2}\tilde{\rho }\tilde{u})=0, \end{array}$$

2.18 $$\begin{array}{rl} & \tilde{\rho }\left(\frac{\partial \tilde{\varphi }}{\partial \tilde{t}}+\tilde{u}\frac{\partial \tilde{\varphi }}{\partial \tilde{r}}\right)=\frac{1}{{\tilde{r}}^{2}}\frac{\partial }{\partial \tilde{r}}\left({\tilde{r}}^{2}\frac{\partial \tilde{\varphi }}{\partial \tilde{r}}\right), \end{array}$$

2.19 $$\begin{array}{rl} & (1+\chi \tilde{\varphi })\tilde{\rho }\left(\frac{\partial \tilde{\theta }}{\partial \tilde{t}}+\tilde{u}\frac{\partial \tilde{\theta }}{\partial \tilde{r}}\right)=\text{Le}\frac{1}{{\tilde{r}}^{2}}\frac{\partial }{\partial \tilde{r}}\left({\tilde{r}}^{2}\frac{\partial \tilde{\theta }}{\partial \tilde{r}}\right)+\chi \frac{\partial \tilde{\varphi }}{\partial \tilde{r}}\frac{\partial \tilde{\theta }}{\partial \tilde{r}} \end{array}$$

2.20 $$\begin{array}{rl} \text{and}\; & 1=\tilde{\rho }(1+\zeta \tilde{\varphi })(1+\kappa \tilde{\theta }), \end{array}$$

where the momentum equation (??) has been omitted since it is decoupled.

By assuming that the liquid phase is pure, incompressible and has constant properties with respect to temperature and that there is no internal convection and that radiative heat transfer is negligible, the liquid phase requires only the energy equation, which becomes

2.21 $$\frac{\partial \tilde{\theta }}{\partial \tilde{t}}=\frac{\text{Le}}{\bar{\alpha }}\frac{1}{{\tilde{r}}^{2}}\frac{\partial }{\partial \tilde{r}}\left({\tilde{r}}^{2}\frac{\partial \tilde{\theta }}{\partial \tilde{r}}\right).$$

**Figure 2.**
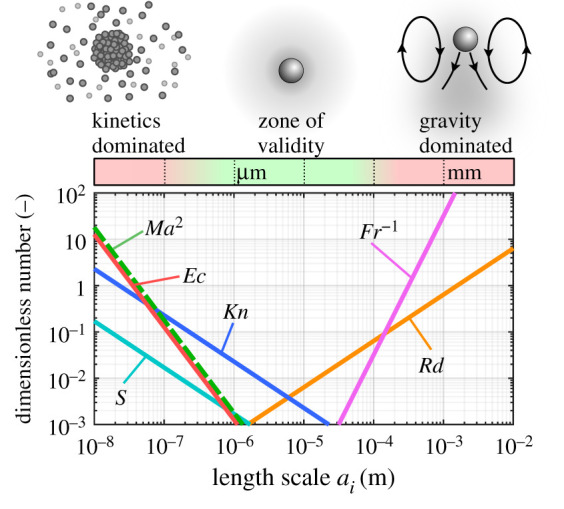
Variation in magnitudes of dimensionless numbers (Mach Ma, Eckert Ec, Knudsen Kn, Froude Fr, radiation number Rd and surface tension number S) with the size of the droplet. Illustrative example for n-octane droplets in air at 600 K and 1 atm. For droplet sizes where all indicated numbers are small, the present spherically symmetric and macroscopic analysis is valid. (Online version in colour.)

It is important to note that all scale-dependent dimensionless numbers (numbers that depend on $a_{i}$) have been eliminated. Therefore, when ${\text{Fr}}^{-1},$ Ec, ${\text{Ma}}^{2}\ll 1$ the system becomes independent of scale. The reduced system is fully described by the 10 governing dimensionless numbers listed in table 1 (except for scale-dependent numbers and Sc). Verifying that the scale-dependent numbers are small is case specific, but figure 2 shows an illustrative example for n-octane droplets in air.

Additionally, although they do not appear in the present governing equations, it must be verified that the numbers Kn, Rd and S are small to justify the continuum assumption, neglecting radiative heat transfer and neglecting surface tension effects, respectively. For small $\text{Kn}=l_{\text{coll}}/a_{i}$, the fluid may be treated as a continuum and molecular effects are negligible. The mean free path length is evaluated at the surface by $l_{\text{coll}}={\left(\sqrt{2}\pi d_{m}^{2}\bar{n}\right)}^{-1}$, where $d_{m}$ is the characteristic molecule diameter and $\bar{n}$ is the molecule number density. Radiative heat transfer effects are negligible for small $\text{Rd}=a\eta \sigma_{\text{SB}}c_{p}^{A}(T_{\infty }^{4}-T_{\text{Q S},s}^{4})/[2\lambda_{\infty }L\ln(B_{T}+1)]$ [26], where $\sigma_{\text{SB}}$ is the Stefan–Boltzmann constant and η is the effective absorption coefficient and is estimated as 0.93. Finally, the increase in surface vapour concentration due to surface tension is negligible for small $\text{S}=2\varsigma /(\rho_{l}R^{A}T_{\text{Q S},s}a)$ [22], where ς is the surface tension. As the example in figure 2 shows, all scale-dependent numbers are small, say $<10^{-1}$, over a range of droplet sizes spanning approximately three orders of magnitude and are $<10^{-2}$ for approximately one order of magnitude for droplet sizes $∼10^{-5}\;\text{m}$. Droplets of n-octane in this range ($∼10^{-5}\;\text{m}$) are of significant practical interest in, for example, fuel sprays for combustion. Each scenario will require individual verification of the validity of these assumptions.

The dimensionless boundary conditions are as follows. Since Fr≫1, spherical symmetry holds and therefore spatial derivatives are zero at $\tilde{r}=0$. The far-field dimensionless temperature and mass fraction are zero, i.e. $\tilde{\theta }$, $\tilde{\varphi }\to 0$ for $\tilde{r}\to \infty$ and the far-field dimensionless pressure becomes unnecessary because incompressibility is assumed. Through the state equation (2.20), this also fixes the dimensionless density at infinity to unity. From insolubility, equation (2.5) becomes

2.22 $${\tilde{j}}_{\text{ev}}\left(1+\frac{1}{B_{M}}-{\tilde{\varphi }}_{s}\right)+\tilde{\Gamma }{\left[\frac{\partial \tilde{\varphi }}{\partial \tilde{r}}\right]}_{s}=0,$$

where ${\tilde{j}}_{\text{ev}}$ is the dimensionless evaporative mass flux, ${\tilde{j}}_{\text{ev}}={\tilde{\rho }}_{s}({\tilde{u}}_{s}-\text{d}\tilde{a}/\text{d}\tilde{t})$, and $\tilde{\Gamma }=1$ under the constant transport property assumption. The rate of change of droplet radius is

2.23 $$\frac{\text{d}\tilde{a}}{\text{d}\tilde{t}}=-\bar{\rho }\;{\tilde{j}}_{\text{ev}}.$$

Surface temperature and energy flux continuity, given by equation (2.7), respectively, become

2.24 $${\tilde{\theta }}_{s}^{-}={\tilde{\theta }}_{s}^{+}\;\text{and}\;{\left[\frac{\partial \tilde{\theta }}{\partial \tilde{r}}\right]}_{s}^{-}=\bar{\lambda }\tilde{\lambda }{\left[\frac{\partial \tilde{\theta }}{\partial \tilde{r}}\right]}_{s}^{+}-\frac{\bar{\lambda }{\bar{c}}_{p}}{\text{Le}B_{T}}{\tilde{j}}_{\text{ev}},$$

where $\tilde{\Gamma },\tilde{\lambda }=1$ assuming constant transport properties. The dimensionless form of equation (2.8) relates the surface temperature (${\tilde{\theta }}_{s}$) to the surface mass fraction (${\tilde{\varphi }}_{s}$) as

2.25 $$\frac{B_{M}\Phi }{B_{M}+1}{\tilde{\varphi }}_{s}+1-\Phi ={\left\{\frac{1+{\text{Ma}}^{2}\tilde{P}}{\varepsilon }\exp\left[\frac{\gamma }{\gamma -1}\left(\frac{1}{\text{Ja}T+B_{T}{\tilde{\theta }}_{s}}-\frac{1}{\text{Ja}}\right)\right]+1-\frac{1}{\varepsilon }\right\}}^{-1},$$

where $1+{\text{Ma}}^{2}\tilde{P}∼1$ under the incompressibility assumption.

### (c) Initial conditions

The required initial conditions are the liquid temperature field ($\tilde{\theta }$ for $\tilde{r}\leq 1$) and the gas temperature and vapour concentration fields ($\tilde{\theta }$, $\tilde{\varphi }$ for $\tilde{r}>1$). The dimensionless approach and assumptions preclude the need to specify the initial droplet size or the pressure and velocity fields.

The present study considers two types of initial conditions, the first of which is referred to as the *step initial conditions* where the liquid is initially at a uniform temperature ${\tilde{\theta }}_{l,i}$ and the gas phase is at uniform temperature and vapour concentration equal to the far-field values, i.e. $\tilde{\theta }={\tilde{\theta }}_{l,i}$ for $\tilde{r}\leq 1$ and $\tilde{\theta },\tilde{\varphi }=0$ for $\tilde{r}>1$. Physically, this is as if the droplet is suddenly immersed into an undisturbed atmosphere. Generally, in the present study, the initial liquid temperature is set equal to the QS value, i.e. ${\tilde{\theta }}_{l,i}=-1$, to isolate the gas phase transient effects. It is important to note that even though the initial liquid temperature may be set to the QS value, this does not mean that the liquid temperature is constant. The imbalance of heat and mass fluxes at the surface can still cause droplet heating or cooling, see equation (2.24). The effect of droplet heating (${\tilde{\theta }}_{l,i}<-1$) is still considered in the context of recovering past solutions, as will be presented in §4.a.

The second type of initial condition is the *QS initial conditions*, where the initial gas phase fields are equal to the QS fields, as will be shown in equation (3.4), and the initial liquid temperature ${\tilde{\theta }}_{l,i}$ is −1. It is important to note that in cases where the QS assumption is valid, both types of initial conditions should give the same results since the initially uniform gas field will rapidly converge to the QS field and the droplet size histories should be near-indistinguishable.

With the initial conditions defined, the problem is a transient heat and mass transfer problem with a moving boundary. The close coupling of the governing equations, the nonlinearity introduced by the ideal gas equation of state and the added complexity of a moving boundary indicates a numerical solution is required. To solve the system of equations accounting for the receding droplet surface, a moving mesh was implemented to ensure that the droplet surface always coincides with the boundary of mesh elements. The governing equations were transformed using Reynolds transport theorem and then discretized. This introduced the purely numerical parameters, $\Delta {\tilde{r}}_{\text{nom}}$ and $\Delta {\tilde{t}}_{\text{nom}}$, which represent the nominal spatial and temporal discretization parameters, respectively. The finite numerical space introduces an additional numerical parameter, ${\tilde{r}}_{\infty }$, which is the size of the domain. It was ensured the obtained solutions were independent of further refinement of the numerical parameters $\Delta {\tilde{r}}_{\text{nom}}$, $\Delta {\tilde{t}}_{\text{nom}}$ and ${\tilde{r}}_{\infty }$. An arbitrarily large value of 1000 was used for ${\tilde{r}}_{\infty }$ meaning that the domain was 1000 times the initial size of the droplet. First-order accuracy for spatial derivatives and explicit time discretization was used. Further details of the discretized equations and solution procedure is given in the electronic supplementary material, S2.

## 3. The QS problem

The QS problem is now summarized for two main reasons; firstly, to provide the definitions of $B_{M}$ and $B_{T}$ which readily derive from the QS solution; and secondly, to define the QS droplet lifetime ${\tilde{t}}_{\text{ev},\text{Q S}}$ which is used as a benchmark to compare with the fully transient model. The full derivation of the QS solution is not presented here but is included in the electronic supplementary material, S3, for completeness. Various equivalent full derivations can be found in the literature [20], but these are not in the present dimensionless framework. Here we present only the key results and discuss the important features of the QS solution.

The full QS problem is obtained by adopting assumptions 1–25 in appendix A. With these assumptions, the heat diffusion into the droplet balances the mass flux leaving the droplet (related through L) such that the liquid temperature remains constant at the wet-bulb temperature, hence ${\tilde{\theta }}_{s}=-1$ and ${\tilde{\varphi }}_{s}=1$. From the QS assumption, time derivatives in the gas phase governing equations (2.17)–(2.21) are zero. Solving the species and energy equation independently yields two expressions for evaporation rate, which can be equated to give the following significant and widely reported result

3.1 $$1+B_{M}={\left(1+B_{T}\right)}^{\text{Le}/{\bar{c}}_{p}}.$$

An additional relationship for $B_{M}$ and $B_{T}$ is obtained from the saturation properties by substituting ${\tilde{\theta }}_{s}=-1$ and ${\tilde{\varphi }}_{s}=1$ into equation (2.25), resulting in

3.2 $$B_{T}=\text{Ja}T-{\left[\frac{1}{\text{Ja}}-\frac{\gamma -1}{\gamma }\ln\left(\frac{1+B_{M}-\Phi }{1+B_{M}+\Phi (\varepsilon -1)}\right)\right]}^{-1}.$$

Solving equations (3.1) and (3.2) simultaneously yields the definition of the dimensionless numbers $B_{T}$ and $B_{M}$ as a function of six dimensionless numbers, $\text{Le}/{\bar{c}}_{p}$, Ja, T, Φ, ε and γ.

**Figure 3.**
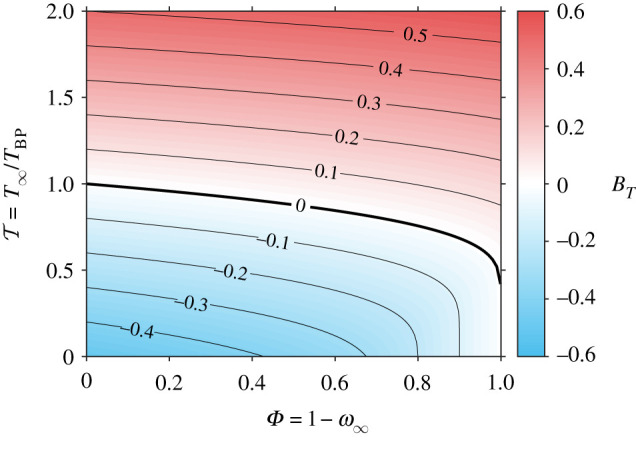
Spalding heat transfer number ($B_{T}$) map as a function of ambient vapour concentration (Φ) and excess temperature ratio (T). Based on constants: Ja=0.5, ε=1, γ=1.66, $\text{Le}/{\bar{c}}_{p}=1$. (Online version in colour.)

For given fluid properties, the numbers $B_{M}$ and $B_{T}$ become a function of the boundary conditions only, as shown in figure 3. In this example, $\text{Le}/{\bar{c}}_{p}=1$ such that $B_{M}=B_{T}$. The saturation line occurs where $B_{T}=0$, meaning that the atmosphere is saturated with vapour and no phase change occurs. For $B_{T}<0$, condensation occurs and for T>1, the ambient temperature is in excess of boiling temperature such that $B_{T}>0$ for all Φ, i.e. evaporation takes place regardless of ambient concentration. When T≫1, the surface temperature approaches the boiling temperature ($T_{\text{Q S},s}\to T_{\text{BP}}$) such that $B_{T}\to \text{Ja}(T-1)$ and $B_{T}$ approaches becoming independent of Φ. In the limit Φ→0, the droplet is immersed in its own vapour and phase change occurs at boiling temperature, meaning that $B_{T}=\text{Ja}(T-1)$.

The QS evaporation rate is

3.3 $$\frac{\text{d}({\tilde{a}}^{2})}{\text{d}\tilde{t}}=-2\bar{\rho }\;\frac{\text{Le}}{{\bar{c}}_{p}}\ln(1+B_{T}),\;\text{therefore}\;{\tilde{t}}_{\text{ev},\text{Q S}}={\left[2\bar{\rho }\;\frac{\text{Le}}{{\bar{c}}_{p}}\ln(1+B_{T})\right]}^{-1},$$

which gives the $d^{2}$-law and the QS droplet lifetime ${\tilde{t}}_{\text{ev},\text{Q S}}$, respectively. The dimensionless QS temperature and concentration fields are respectively

3.4 $${\tilde{\theta }}_{\text{Q S}}=-\frac{B_{T}+1}{B_{T}}\left[1-{\left(\frac{1}{B_{T}+1}\right)}^{\tilde{a}/\tilde{r}}\right]\;\text{and}\;{\tilde{\varphi }}_{\text{Q S}}=\frac{B_{M}+1}{B_{M}}\left[1-{\left(\frac{1}{B_{M}+1}\right)}^{\tilde{a}/\tilde{r}}\right].$$

Hence, if $B_{T}$ and $B_{M}$ are equal, the temperature and mass fields are equal but opposite. For small $B_{T}$, the temperature profile approaches a pure conduction $r^{-1}$ profile (${\tilde{\theta }}_{\text{Q S}}=-\tilde{a}/\tilde{r}$). As $B_{T}$ increases, the *blowing* effect (i.e. increased Stefan velocity) leads to a flatter profile near the droplet surface that partially attenuates the evaporation process.

Three key features of the QS solution are identified. Firstly, the evaporation process is history independent since the evaporation rate does not depend on the initial droplet size. This implies a given droplet may be observed at any arbitrary time at which the initial size can be defined. Secondly, the concentration and temperature fields are self-similar with space scale, i.e. they scale down in proportion with the droplet size since the field is only a function of $\tilde{r}/\tilde{a}$. Finally, the amount of vapour contained within the field is infinite and the amount of energy required to cool down the atmosphere to establish the temperature profile is also infinite (further details in electronic supplementary material, S4). This is a logical inconsistency since the amount the mass within the droplet and the energy that the drop can absorb is finite.

Liquid phase transients can be considered in conjunction with a QS gas phase by retaining the time derivative in equation (2.21) as reported in a few notable works [22,29,37] as well as the present study in §4.a. In this model, the droplet surface temperature begins at an initial value ${\tilde{\theta }}_{s,i}$ and then converges to the QS value (−1), at which point the droplet temperature remains constant. The full QS solution is therefore a special case where ${\tilde{\theta }}_{s,i}=-1$. Generally, in this study, liquid phase transients are minimized by setting the initial droplet temperature to the QS temperature such that the gas phase transient effects can be isolated.

## 4. Results of quasi-stationary and fully transient models

The key results of the present investigation are now presented. Firstly, the present model is validated through recovering solutions of past results by Hubbard *et al.* [30]. This is followed by presenting results from the *quasi-stationary* model. The quasi-stationary model is distinct from the QS model and can be seen as an intermediate model between the QS and fully transient approaches. The QS approach considers a steady gas phase and a stationary droplet surface, whereas the quasi-stationary approach considers a transient gas phase but still with a stationary droplet surface and the fully transient approach considers a transient gas phase with a moving droplet surface. The quasi-stationary model is included as a semi-analytical approach to aid understanding of the effects of gas phase transients. As a significant result of the quasi-stationary model, a key parameter emerges, referred to as delta (δ) in the following. It was found that δ is also important for predicting the outcome of the fully transient model.

### (a) Recovering past results

**Figure 4.**
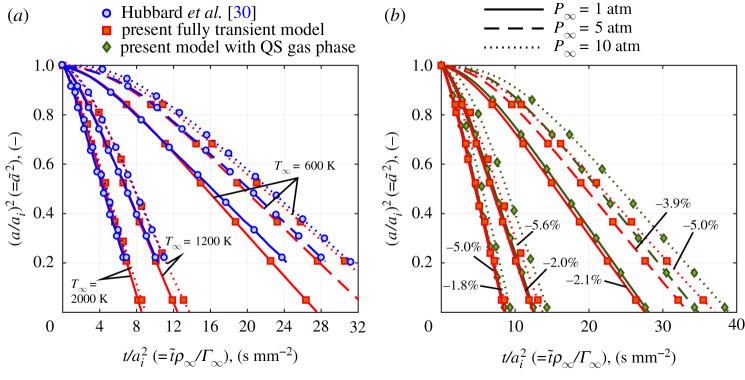
Droplet size histories for octane droplets vaporizing in air at various temperatures and pressures. (*a*) Present fully transient model compared with Hubbard *et al.* [30]; (*b*) present fully transient model compared with the quasi-steady (QS) model; % values indicate difference in total lifetime prediction compared with the QS model. (Online version in colour.)

Figure 4*a* shows results from the present fully transient model compared with those from Hubbard *et al.* [30]. These results consider octane vaporizing in air at various temperatures and pressures. Results from [30] were obtained with fully variable properties, while properties are constants in the present model and are evaluated at a representative film temperature, given by $T_{f}=T_{\text{BP}}+(T_{\infty }-T_{\text{BP}})/3$, which leads to small differences. While the presented size histories match extremely closely, fine tuning of the film temperature can give a more precise match. For example, once the droplet reaches its steady temperature, the approximately constant $d^{2}$-law gradient is sensitive to transport properties. The full list of parameters that describe the system for all of the pressure and temperature conditions is given in the electronic supplementary material, along with the code to reproduce these results.

The present model is advanced to much smaller droplet sizes (${\tilde{a}}^{2}=0.0001$ in this case) rather than terminating at ${\tilde{a}}^{2}∼0.2$. To the present authors’ knowledge, terminating at ${\tilde{a}}^{2}∼0.2$ is a limitation of all reported fully transient studies to date that address the present problem. The present model investigates virtually the full droplet lifetime. The present authors’ findings generally agree with the conclusion of [30] that, in these specific cases, gas phase transients have a small effect. Figure 4*b* compares the fully transient results with those obtained with a QS gas phase for the same conditions. The QS gas phase model (finite conductivity model) still features transient conduction in the liquid phase. The present work has gone further to quantify the differences as indicated by percentages in figure 4*b*. It appears that the differences increase with ambient pressure but are relatively insensitive to ambient temperature.

### (b) The fixed diameter problem and the quasi-stationary model

This section provides insight into how the gas phase responds to the sudden immersion of an evaporating droplet. In the special case where $\bar{\rho }=0$, the droplet diameter is fixed and, by definition, the droplet lifetime is infinite. In the further special case when convection from the droplet surface is small (e.g. small $B_{T}$) then a pure conduction case emerges, for which an analytical solution exists. For a fixed diameter ($a=a_{i}$) sphere of fixed temperature ($T_{\text{Q S},s}$) immersed in a gas of initially uniform temperature ($T_{\infty }$), the temperature gradient at the surface ${\left[\nabla T\right]}_{s}$ develops as [38]

4.1 $${\left[\nabla T\right]}_{s}=\frac{T_{\infty }-T_{\text{Q S},s}}{a_{i}}\left[1+\frac{a_{i}}{{\left(\pi \;\alpha \;t\right)}^{1/2}}\right],\;\text{or equivalently}\;{\left[\tilde{\nabla }\tilde{\theta }\right]}_{s}=1+\frac{1}{{\left(\pi \;\text{Le}\;\tilde{t}\right)}^{1/2}}.$$

We define Λ as the fractional difference between the instantaneous surface temperature gradient and the steady surface temperature gradient, i.e.

4.2 $$\Lambda =\frac{{\left[\tilde{\nabla }\tilde{\theta }\right]}_{s}}{{\left[\tilde{\nabla }\tilde{\theta }\right]}_{\text{Q S},s}}-1,\;\text{where}\;{\left[\tilde{\nabla }\tilde{\theta }\right]}_{\text{Q S},s}=\frac{\ln(1+B_{T})}{B_{T}\tilde{a}}\;\text{from equation (3.4).}$$

Therefore, for the pure conduction case ($B_{T}\to 0$), $\Lambda ={\left(\pi \;\text{Le}\;\tilde{t}\right)}^{-1/2}$. The temperature gradient at the surface is significant in the context of evaporation since this determines the evaporation rate for a steady surface temperature. The parameter Λ can be seen as a time-dependent correction to the surface temperature gradient due to gas phase transients.

**Figure 5.**
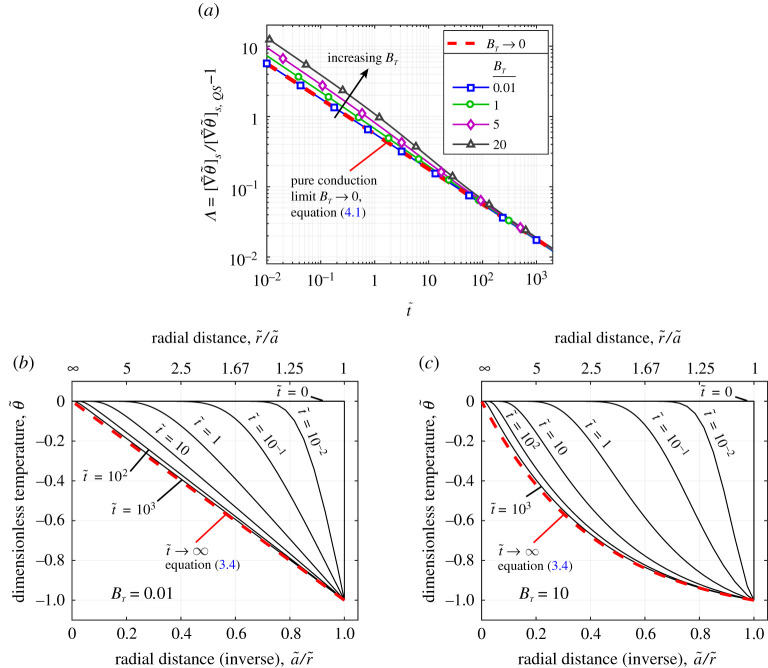
Results for fixed diameter droplet ($\bar{\rho }=0$). (*a*) Transient surface temperature gradient as fractional difference from quasi-steady value (Λ) for a range of $B_{T}$ values. (*b* and *c*) Gas phase temperature profile at various times for small and large values of $B_{T}$, respectively ($B_{T}=0.01,\;10$). Constants: Ja=0.5, γ=1.4, ${\bar{c}}_{p}=1$, ε=1, Φ=1, Le=1. (Online version in colour.)

Figure 5*a* shows the time development of the surface temperature gradient for various evaporative fluxes obtained from the present fully transient model with $\bar{\rho }=0$. Increasing $B_{T}$ indicates increased evaporative flux and hence increased convective velocity. Firstly, in the small $B_{T}$ limit, it can be seen that the present model precisely recovers the analytical solution for pure conduction. At elevated $B_{T}$, there is initially a greater fractional deviation from the steady value compared with the pure conduction limit. As time advances, the elevated $B_{T}$ cases converge closely towards the pure conduction case. A key finding here is that there is relatively little difference between the convergence time for the high and low $B_{T}$ cases.

Figure 5*b*,*c* shows how the temperature field develops over time for small and relatively large values of $B_{T}$. The increased convective flow in the large $B_{T}$ case causes more rapid temperature changes in the region close to the droplet. However, because the steady temperature profile in the region surrounding the droplet is much flatter, the convergence towards the QS field is slower at elevated $B_{T}$. This is the reason for the greater initial deviation (in fractional terms) of the temperature gradient as $B_{T}$ increases in figure 5*a*.

The temporal development of the surface temperature gradient in this stationary case can be used as a first approximation to determine how the gas phase transients will affect the evaporation process. The time scale for droplet evaporation is given by equation (3.3) and if this time is comparable with the convergence time of the surface temperature gradient, then transient effects should be expected to manifest. For example, if the dimensionless evaporation time is approximately 10 then this quasi-stationary approach predicts that the surface gradient will, for all the droplet lifetime, be 10% greater in magnitude than the QS value. Conversely, if the dimensionless evaporation time is approximately $10^{4}$ then, for the majority of the droplet’s lifetime, the surface temperature gradient will be within 1% of the QS value. In terms of quantitatively predicting how this will effect the entire droplet lifetime, one can construct a quasi-stationary model. This semi-analytical approach assumes that the surface temperature gradient develops as if the droplet size is fixed. This is accomplished as follows.

Combining equations (2.22)–(2.24) for fixed surface conditions (${\tilde{\varphi }}_{s}=1$) leads to

4.3 $$\frac{\text{d}\tilde{a}}{\text{d}\tilde{t}}=-\bar{\rho }\frac{\text{Le}}{{\bar{c}}_{p}}B_{T}{\left[\tilde{\nabla }\tilde{\theta }\right]}_{s},$$

which combined with (4.2) gives

4.4 $$\frac{\text{d}({\tilde{a}}^{2})}{\text{d}\tilde{t}}=-\frac{1}{{\tilde{t}}_{\text{ev},\text{Q S}}}(1+\Lambda ).$$

For small $B_{T}$, then $\Lambda ={\left(\pi \;\text{Le}\;\tilde{t}\right)}^{-1/2}$, and equation (4.4) can be integrated to give the quasi-stationary evaporation time ${\tilde{t}}_{\text{ev},Q\text{stat}}$ as

4.5 $${\tilde{t}}_{\text{ev},Q\text{stat}}={\tilde{t}}_{\text{ev},\text{Q S}}+\frac{2}{\pi \text{Le}}[1-{\left(1+\pi \;\text{Le}\;{\tilde{t}}_{\text{ev},\text{Q S}}\right)}^{1/2}].$$

Defining δ as the fractional difference between the predicted evaporation time from the quasi-stationary model and the full QS model, i.e.

4.6 $$\delta =\frac{{\tilde{t}}_{\text{ev},Q\text{stat}}}{{\tilde{t}}_{\text{ev},\text{Q S}}}-1,\;\text{then}\;\delta =\frac{2}{\pi \;\text{Le}\;{\tilde{t}}_{\text{ev},\text{Q S}}}[1-{\left(1+\pi \;\text{Le}\;{\tilde{t}}_{\text{ev},\text{Q S}}\right)}^{1/2}],$$

which, for $\pi \;\text{Le}\;{\tilde{t}}_{\text{ev},\text{Q S}}\gg 1$, reduces to the final expression for δ as

4.7 $$\delta =-{\left[\frac{8\bar{\rho }\ln(1+B_{T})}{\pi {\bar{c}}_{p}}\right]}^{1/2}.$$

**Figure 6.**
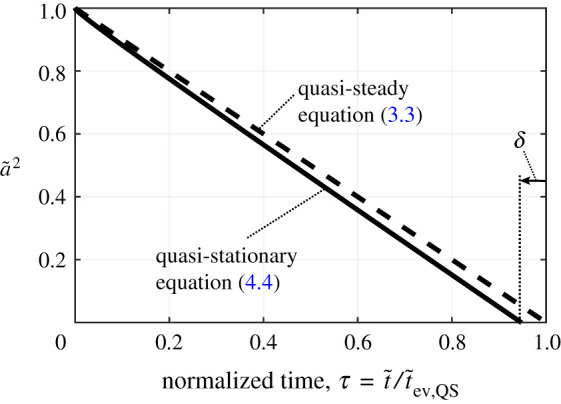
Example droplet size history comparison between the quasi-steady ($d^{2}$-law) and the quasi-stationary model. Constants: $\bar{\rho }=0.004$, $B_{T}=0.3162$, ${\bar{c}}_{p}=1$.

This gives a first approximation for quantifying the deviation from QS evaporation caused by an initially undisturbed atmosphere, i.e. the step initial conditions. In the early stages of droplet lifetime, faster evaporation results from large temperature and concentration gradients at the surface that take time to converge towards the steady value because of the thermal and mass inertia introduced by the atmosphere. Figure 6 shows an example of the droplet size development predicted by this model and shows graphically what the quantity δ represents. In this case, the full numerical solution gives δ=−0.0525, while equation (4.7) gives δ=−0.0529. The small difference is due to the approximations in deriving equation (4.7) such as small $B_{T}$.

It should be stressed that the quasi-stationary model is purely an initial approximation for quantifying the effects of gas phase transients since the convergence of the surface temperature gradient was formed based on a fixed diameter droplet. However, the trends captured by equation (4.7) are extremely informative. The fully transient model goes further to investigate the dynamic effects of a moving boundary.

### (c) Characterizing fully transient evaporation dynamics

The previous quasi-stationary model does not capture the effects of a moving boundary in terms of the convergence of the gas phase towards the steady field and the analysis breaks down for large $B_{T}$. It is therefore desired to develop a complete understanding of gas phase transients in conjunction with a moving boundary and to quantify the differences between the fully transient model and the QS model for a wide variety of conditions. This section first presents some illustrative examples in order to define the transient deviation criteria used and to present and explain typical evaporation dynamics observed. The next section then generalizes these findings for a wide range of conditions by exploring how each of the governing dimensionless numbers influence the gas phase transients.

**Figure 7.**
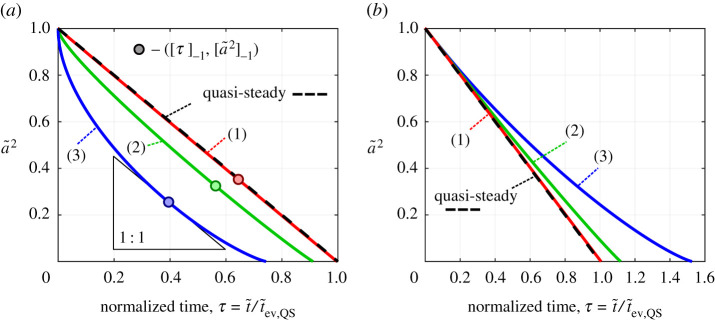
Droplet radius (squared) versus normalized time for three cases, (1), (2) and (3). Results from the fully transient model (*a*) with step initial conditions, (*b*) with quasi-steady (QS) initial conditions. The dots indicate the coordinates where the gradient is −1. (Online version in colour.)

Figure 7 shows three example droplet size histories that deviate from the QS case to varying degrees. The *x*-axis is rescaled with respect to the QS evaporation time, $\tau =\tilde{t}/{\tilde{t}}_{\text{ev},\text{Q S}}$, such that all cases follow the same $d^{2}$-law path if the QS assumption is applied. The inputs for each of the three cases are given in table 2. To quantify the deviation from QS behaviour, three parameters are now introduced and are defined as

4.8 $$\epsilon_{\text{net}}=\tau_{\text{ev}}-1,\;\epsilon_{\text{start}}=\frac{{\left[\tau \right]}_{-1}}{1-{\left[{\tilde{a}}^{2}\right]}_{-1}}-1\;\text{and}\;\epsilon_{\text{end}}=\frac{\tau_{\text{ev}}-{\left[\tau \right]}_{-1}}{{\left[{\tilde{a}}^{2}\right]}_{-1}}-1,$$

where $\tau_{\text{ev}}$ is the normalized evaporation time and the point (${\left[\tau \right]}_{-1},{\left[{\tilde{a}}^{2}\right]}_{-1}$) is defined as the point where the transient gradient equals the QS gradient, i.e. $\text{d}({\tilde{a}}^{2})/\text{d}\tau =-1$. In figure 7*a*, this is the point of maximum deviation from the QS profile. The parameter $\epsilon_{\text{net}}$ effectively describes the overall difference in the total lifetime of the droplet, while $\epsilon_{\text{start}}$ and $\epsilon_{\text{end}}$ collectively describe the shape of the profile. If all ϵ parameters are zero, then the fully transient solution equals the QS solution. The regimes separated by ${\left[\tau \right]}_{-1}$ are distinct in that the initial evaporation rate is greater than that predicted by the QS model ($\epsilon_{\text{start}}<0$), while the evaporation rate towards the end of life is slower than that predicted by the QS model ($\epsilon_{\text{end}}>0$). In the presented cases (and in most cases), this transition ${\left[{\tilde{a}}^{2}\right]}_{-1}$ occurs at approximately ${\tilde{a}}^{2}∼0.3$, equivalent to when the droplet is approximately half its original diameter. Therefore, failure to advance the solution past ${\tilde{a}}^{2}∼0.2$ will not capture the dynamic evaporation behaviour observed. To the present authors’ knowledge, the evaporation dynamics towards the end of life have not yet been described and explained in the available literature. They do, however, match qualitatively with results presented when only transient mass transport was considered in conjunction with a moving boundary [39].

**Table 2.** Transient deviation parameters ($\epsilon_{\text{start}}$, $\epsilon_{\text{end}}$, $\epsilon_{\text{net}}$) calculated for various cases. Inputs kept constant for all cases are: Le=1, Φ=1, ${\bar{c}}_{p}=1$, ε=1, γ=1.4, $\bar{\lambda }=0.2$, $\bar{c}=0.5$, ${\tilde{\theta }}_{l,i}=-1$.

			step initial conditions			QS initial conditions		
	inputs	$B_{T}$	$\epsilon_{\text{start}}$	$\epsilon_{\text{end}}$	$\epsilon_{\text{net}}$	$\epsilon_{\text{start}}$	$\epsilon_{\text{end}}$	$\epsilon_{\text{net}}$
(1)	$\bar{\rho }=1\times 10^{-3}$	0.018	−0.006	0.002	−0.003	0	0.004	0.004
	T=0.9, Ja=0.2							
(2)	$\bar{\rho }=20\times 10^{-3}$	1.04	−0.167	0.076	−0.089	0	0.116	0.116
	T=3, Ja=0.5							
(3)	$\bar{\rho }=80\times 10^{-3}$	10.2	−0.468	0.370	−0.256	0	0.525	0.525
	T=5, Ja=2.5							

Considering figure 7*a*, the three cases differ significantly in their deviation from QS evaporation. Case (1) is essentially indistinguishable from QS evaporation, i.e. QS evaporation has been recovered by the model in the limit of small $B_{T}$. This case resembles what would be seen, for example, when water evaporates under atmospheric conditions. Case (2) exhibits notable deviation from QS evaporation where the majority of the deviation occurs in the initial stages. One could conceivably interpret this profile as being linear (ignoring the initial period) if this size history was measured, despite the curve. Case (3), however, departs significantly from QS evaporation and shows a very distinctive curve of decreasing gradient resulting in a factor ∼2 difference in the time to reach ${\tilde{a}}^{2}∼0.25$ and an overall 26% difference in total droplet lifetime.

**Figure 8.**
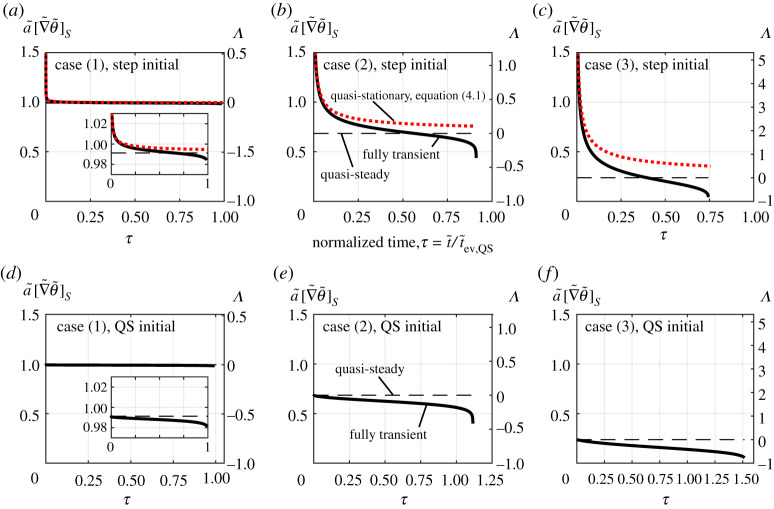
Normalized surface temperature gradient histories based on the quasi-steady (QS) model (dashed), quasi-stationary model (dotted) and the fully transient model (solid), (*a*–*c*) with step initial conditions and (*d*–*f* ) with QS initial conditions. The temperature gradient relative to the QS value (Λ) is also shown. Three cases shown with input values given in table 2. (Online version in colour.)

Figure 8 presents the surface temperature gradient development with time for the selected three cases and provides insight into why the size histories of figure 7 are observed. A key observation is that once the temperature gradient converges towards the steady value with step initial conditions (figure 8*a*–*c*), it does not remain constant as may be implied by the term *steadiness*. The temperature gradient instead diverges from the steady value towards the end of life. This is because the cooled and saturated boundary layer established during the early stages of evaporation can remain and insulate the droplet during the late stages of evaporation. Therefore, the environment experienced by the droplet towards the end of life is of lower temperature and is more saturated than suggested by $T_{\infty }$ and $\omega_{\infty }$ leading to slower evaporation rates. It was found that this *self-insulating* effect can lead to the droplet temperature heating or cooling towards the end of life. Whether the droplet heats or cools depends on the quantity $\text{Le}/{\bar{c}}_{p}$, where the droplet temperature increases for $\text{Le}/{\bar{c}}_{p}>1$ and *vice versa*. Figure 8*a*–*c* also shows the transient temperature gradient as predicted by the quasi-stationary model. The quasi-stationary model matches the fully transient model in the early stages when ${\tilde{a}}^{2}∼1$ since these are the conditions under which the quasi-stationary model is derived. Following this, the fully transient model predicts a much more rapid convergence towards the steady value of surface temperature gradient. Since the steady surface temperature gradient increases as the droplet shrinks, see equation (4.2), the reducing droplet size assists in more rapid convergence. Additionally, the quasi-stationary model does not predict the reversal and divergence from the QS value. This dynamic behaviour is only predicted by full solution and by implementing a moving boundary.

Figures 7*b* and 8*d*–*f* show the effect of the QS initial conditions for the same inputs. With the QS initial conditions, by definition, ${\left[\tau \right]}_{-1}=0$ because the initial gradient is the QS gradient and therefore the initial transient regime is eliminated ($\epsilon_{\text{start}}=0$). However, a key observation is that the second transient regime remains. The cooled and saturated boundary layer insulates the droplet and reduces the evaporation rate. The occurrence of this self-insulating behaviour is therefore independent of initial conditions, while the exact magnitude of the deviation ($\epsilon_{\text{end}}$) is still affected by the initial conditions.

The two types of initial conditions considered are the two possible extremes. The step initial conditions represent the case where there is initially zero influence of the droplet on the surrounding atmosphere, i.e. sudden immersion, while the QS initial conditions represent the case where the droplet has established an infinite boundary layer ($\tilde{\theta }$ and $\tilde{\varphi }$ vary continuously up to an infinite distance). The infinite boundary layer of cooled and saturated gas with the QS initial conditions is more effective at insulating and slowing evaporation compared with the finite boundary layer established at ${\left[\tau \right]}_{-1}$ with the step initial conditions. It is for this reason that $\epsilon_{\text{end}}$ is always larger with the QS initial conditions.

Under conditions where the QS assumption is valid, the solution is essentially independent of initial conditions. This is evidenced by case (1) in figure 7. With varying initial conditions, the parameter $\epsilon_{\text{end}}$ does remain somewhat comparable. However, under conditions where gas phase transients are significant, there is a large difference in the total droplet lifetime prediction ($\tau_{\text{ev}}$) depending on the initial conditions. This carries important implications for experiments, since when conditions are present where gas phase transients become significant, the droplet size history depends on the full gas phase history, which is a challenge to quantify.

To assist understanding, videos are provided in the electronic supplementary material that animate the process of droplet evaporation for each of the three discussed cases with both types of initial conditions.

### (d) Quantifying deviations from the classical QS solution

The previous section used illustrative examples to describe the fully transient evaporation dynamics and to provide the metrics to quantify deviations from QS evaporation ($\epsilon_{\text{start}}$, $\epsilon_{\text{end}}$ and $\epsilon_{\text{net}}$, collectively referred to as ϵ). This section quantifies the deviation in the general case to understand and define the bounds under which the QS assumption obtains reasonable approximation to the fully transient solution. This is achieved by quantifying how $\epsilon_{\text{start}}$, $\epsilon_{\text{end}}$ and $\epsilon_{\text{net}}$ vary with changes to the governing dimensionless numbers and finding conditions under which they are acceptably small. The physical understanding obtained is used to produce a predictive tool for calculating the three ϵ parameters. The remainder of this study proceeds with the step initial conditions.

**Figure 9.**
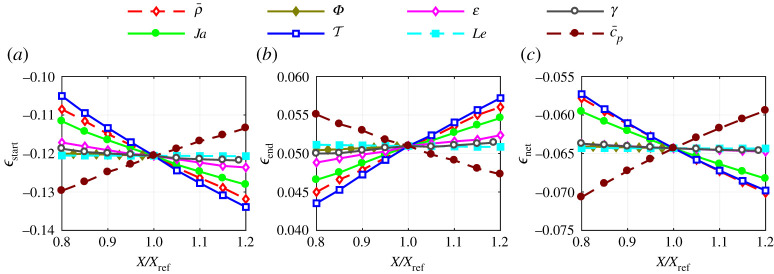
(*a*–*c*) Local sensitivity of transient deviation parameters ($\epsilon_{\text{start}}$, $\epsilon_{\text{end}}$, $\epsilon_{\text{net}}$) to each dimensionless governing number centred about the reference case : $\bar{\rho }=10\times 10^{-3}$, T=3, Ja=0.5, Le=1, Φ=1, ${\bar{c}}_{p}=1$, ε=1, γ=1.4, $\bar{\lambda }=0.2$, $\bar{c}=0.5$. For each dimensionless number, the change relative to the reference case is denoted by $X/X_{\text{ref}}$. (Online version in colour.)

Figure 9 shows the local sensitivity of $\epsilon_{\text{start}}$, $\epsilon_{\text{end}}$ and $\epsilon_{\text{net}}$ to the various governing dimensionless numbers. The significance of gas phase transients is sensitive to, approximately in order of significance, T, $\bar{\rho }$, ${\bar{c}}_{p}$, Ja and ε. The result has a low sensitivity to Le, γ and Φ. An even lower sensitivity is seen to $\bar{c}$ and $\bar{\lambda }$ such that these are omitted from the figure for clarity.

It was hypothesized that the gas phase transient effects are determined primarily by how closely the QS vapour concentration and temperature fields can be approximated by the droplet releasing mass and absorbing heat, respectively. By comparing the amount of vapour contained in the QS field with the amount of mass available in the droplet, one can determine if the droplet is capable of approximating the QS field and, therefore, if the QS assumption is appropriate. To quantify these effects, the parameters ${\tilde{R}}_{M}$ and ${\tilde{R}}_{E}$ are now defined. Here, we provide only the main points, but further details are given in the electronic supplementary material, S4. Defining $R_{M}$ as the radial distance at which the sum of the mass of vapour in the initial QS field equals the mass of vapour available (i.e. the mass of the droplet), then we can write

4.9 $$\rho_{l}V_{i}=4\pi \int_{a_{i}}^{R_{M}}\rho_{\text{Q S}}\left(\frac{\omega_{\text{Q S}}-\omega_{\infty }}{1-\omega_{\infty }}\right)r^{2}\;\text{d}r,$$

where $V_{i}=(4\pi /3)\;a_{i}^{3}$ is the initial droplet volume and the l.h.s. is the mass of the droplet and the r.h.s. represents the sum of vapour in the QS concentration field between the droplet surface and $R_{M}$. The dimensionless equivalent ${\tilde{R}}_{M}$ is defined by

4.10 $$\frac{1}{3\bar{\rho }}=\frac{B_{M}}{B_{M}+1}\int_{1}^{{\tilde{R}}_{M}}{\tilde{\rho }}_{\text{Q S}}{\tilde{\varphi }}_{\text{Q S}}{\tilde{r}}^{2}\;\text{d}\tilde{r},$$

where ${\tilde{\varphi }}_{\text{Q S}}$ is the QS concentration field, as given by equation (3.4), and ${\tilde{\rho }}_{\text{Q S}}$ is the QS density field, obtained by substituting equation (3.4) into equation (2.20). If ${\tilde{R}}_{M}$ is small (close to unity), there is insufficient mass within the droplet to even approximate the initial QS vapour concentration field. However, if ${\tilde{R}}_{M}$ is large then the droplet mass is large compared with the vapour mass surrounding the droplet and, by releasing a small fraction of the droplet mass, the vapour field surrounding the droplet can be established or closely approximated.

Similarly, defining $R_{E}$ as the radial distance at which the energy required to be removed from the atmosphere to establish the QS temperature field $T_{\text{Q S}}$ equals the amount of energy that the droplet is capable of absorbing, then

4.11 $$\rho_{l}V_{i}[L+c_{p}^{A}(T_{\infty }-T_{\text{Q S},s})]=4\pi c_{p,\infty }T_{\infty }\int_{a_{i}}^{R_{E}}\left(\frac{P_{\infty }}{R_{\infty }T_{\text{Q S}}}-\rho_{\infty }\right)r^{2}\;\text{d}r,$$

where the l.h.s. is the energy required to bring the droplet into equilibrium with its environment and the r.h.s. is the heat required to establish the QS temperature field in the ambient gas between the droplet surface and $R_{E}$. The dimensionless equivalent ${\tilde{R}}_{E}$ is defined by

4.12 $$\frac{{\bar{c}}_{p}(B_{T}+1)}{3\bar{\rho }\text{Ja}T}=\int_{1}^{{\tilde{R}}_{E}}\left(\frac{1}{1+\kappa {\tilde{\theta }}_{\text{Q S}}}-1\right){\tilde{r}}^{2}\text{d}\;\tilde{r}.$$

Expanding expressions for ${\tilde{\varphi }}_{\text{Q S}}$ and ${\tilde{\rho }}_{\text{Q S}}$ at an infinite distance up to the first order in $\tilde{r}$ and substituting into equation (4.10) yields the leading order expression for ${\tilde{R}}_{M}$ as

4.13 $${\tilde{R}}_{M}^{\left(0\right)}={\left[\frac{2}{3\bar{\rho }\ln(1+B_{M})}+1\right]}^{1/2}={\left[\frac{16}{3\pi \text{Le}\;\delta^{2}}+1\right]}^{1/2},$$

where superscript (0) indicates the leading order and the expression is simplified by recalling δ, as given by equation (4.7). Similarly, expanding ${\tilde{\theta }}_{\text{Q S}}$ at infinity up to the first order in $\tilde{r}$ and substituting into equation (4.12) gives the leading-order expression for ${\tilde{R}}_{E}$ as

4.14 $${\tilde{R}}_{E}^{\left(0\right)}={\left[\frac{2{\bar{c}}_{p}}{3\bar{\rho }\ln(1+B_{T})}+1\right]}^{1/2}={\left[\frac{16}{3\pi \delta^{2}}+1\right]}^{1/2}.$$

The key finding from equations (4.13) and (4.14) is that at the leading order ${\tilde{R}}_{M}^{\left(0\right)}$ is a function of Le and δ, while ${\tilde{R}}_{E}^{\left(0\right)}$ is a function of only δ. Therefore, for large ${\tilde{R}}_{E}$, or equivalently small δ, the dimensionless combination parameter δ alone quantifies the relationship between the energy that can be absorbed by the droplet and the energy removed to establish the steady temperature field. This is significant because δ was derived in a separate framework (the quasi-stationary model) yet it emerges again in this context as a measure of relating the energy contained within the droplet to the energy required to cool the surrounding atmosphere.

**Figure 10.**
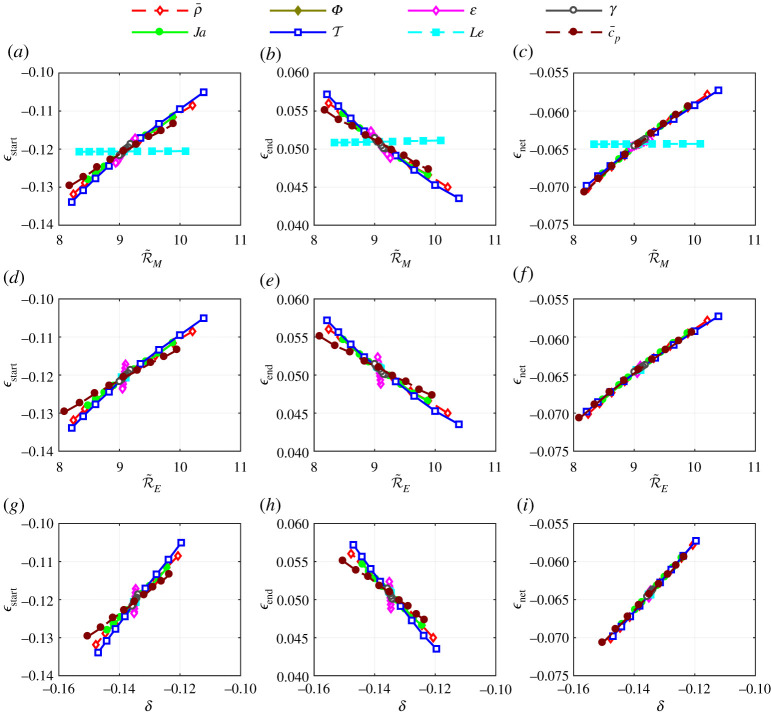
(*a*–*i*) Local sensitivity of transient deviation parameters ($\epsilon_{\text{start}}$, $\epsilon_{\text{end}}$, $\epsilon_{\text{net}}$) in terms of dimensionless combination variables (${\tilde{R}}_{M}$, ${\tilde{R}}_{E}$, δ). Each line represents changing the indicated dimensionless number relative to the specific case with values: $\bar{\rho }=10\times 10^{-3}$, T=3, Ja=0.5, Le=1, Φ=1, ${\bar{c}}_{p}=1$, ε=1, γ=1.4, $\bar{\lambda }=0.2$, $\bar{c}=0.5$. (Online version in colour.)

Figure 10 shows the local sensitivity plot as shown in figure 9, but in terms of the dimensionless combination variables ${\tilde{R}}_{M}$, ${\tilde{R}}_{E}$ and δ. Note that ${\tilde{R}}_{M}$, ${\tilde{R}}_{E}$ and δ are quantified using equations (4.10), (4.12) and (4.7), respectively. While figure 9 shows a complex 10-variable problem (with eight variables shown), the quantities ${\tilde{R}}_{M}$, ${\tilde{R}}_{E}$ and δ remove much of the variance and distil the problem to closely depend on just these combination variables. Figure 10 shows that ${\tilde{R}}_{M}$ closely captures the effect of all dimensionless numbers on the ϵ parameters, with the exception of Le. Each of the lines, which correspond to locally varying one dimensionless input, closely collapse onto a single trendline. This is particularly evident in the case of $\epsilon_{\text{net}}$, while the shape parameters $\epsilon_{\text{start}}$ and $\epsilon_{\text{end}}$ show some deviation even though the general trend is captured. The inability of ${\tilde{R}}_{M}$ to capture the variance due to changes in Le means that Le requires special treatment in order to use ${\tilde{R}}_{M}$ as a predictor of $\epsilon_{\text{start}}$, $\epsilon_{\text{end}}$ and $\epsilon_{\text{net}}$. Similarly, ${\tilde{R}}_{E}$ and δ capture the $\epsilon_{\text{net}}$ trend but are less capable of capturing how changes to the inputs affect $\epsilon_{\text{start}}$ and $\epsilon_{\text{end}}$. In particular, figure 10*d*,*e*,*g* and *h* shows that the variance due to changes in molecular mass ratio ε is not correctly captured. The suspected reason for this is the added complexities introduced by the density dependence on composition as ε deviates from unity, see equation (2.20).

**Figure 11.**
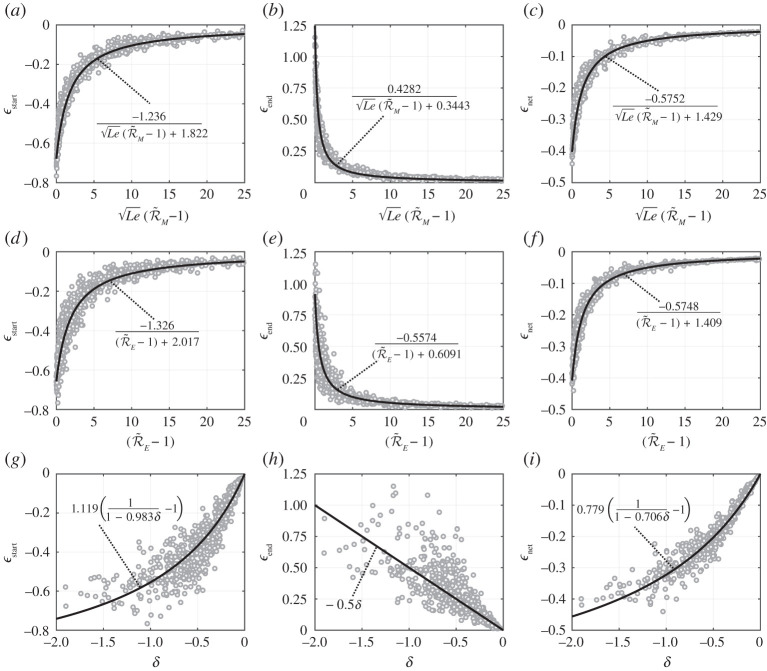
(*a*–*i*) Transient deviation parameters ($\epsilon_{\text{start}}$, $\epsilon_{\text{end}}$, $\epsilon_{\text{net}}$) plotted in terms of dimensionless combination variables (${\tilde{R}}_{M}$ and Le, ${\tilde{R}}_{E}$, δ) for a wide range of inputs. Each point represents results from the fully transient model for different sets of inputs. Lines are curves fitted to data with the equations shown.

This local sensitivity example explores only a narrow region of the possible 10-dimensional input domain, but is informative in terms of the role of each governing dimensionless number. It was desired to test the robustness of ${\tilde{R}}_{M}$, ${\tilde{R}}_{E}$ and δ as predictors of $\epsilon_{\text{start}}$, $\epsilon_{\text{end}}$ and $\epsilon_{\text{net}}$. This was performed by evaluating the ϵ parameters over a wide range of inputs with the fully transient model and plotting them against the corresponding ${\tilde{R}}_{M}$, ${\tilde{R}}_{E}$ and δ values, as shown in figure 11. The range of inputs considered are given in table 3. Each data point, therefore, corresponds to a random, unique set of inputs within the bounds defined in table 3. The limits given in table 3 are informed by typical values seen in practice.

**Table 3.** Upper and lower bounds for input domain in obtaining figure 11.

input variable	(lower bound, upper bound)	input variable	(lower bound, upper bound)
T	[1,10]	Le	[0.25,4]
$\bar{\rho }$	$\left[2\times 10^{-4},0.3\right]$	γ	[1.01,1.66]
${\bar{c}}_{p}$	[0.25,4]	Φ	[0.1,1]
Ja	[0.1,10]	$\bar{c}$	[0.1,1.5]
ε	[0.2,5]	$\bar{\lambda }$	[0,1]

The results presented in figure 11 are the key findings of this study. The trends captured show that fully transient evaporation dynamics can be closely predicted by the readily calculable parameters ${\tilde{R}}_{M}$, ${\tilde{R}}_{E}$ and δ. The wide envelope of inputs considered covers a large range of cases, e.g. ${\tilde{R}}_{E}$ varies from 1.06 to 195. Note that as ${\tilde{R}}_{M}$ or ${\tilde{R}}_{E}$ tend towards unity, the utility of equations (4.13) and (4.12), respectively, diminishes and higher-order effects come into significance.

**Figure 12.**
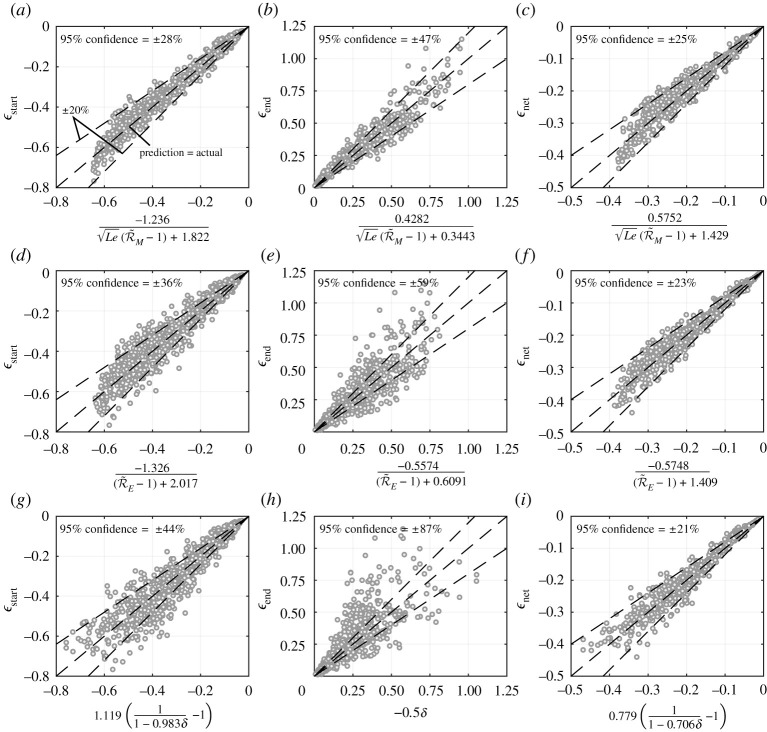
Transient deviation parameters ($\epsilon_{\text{start}}$, $\epsilon_{\text{end}}$, $\epsilon_{\text{net}}$) taken from the fully transient model (y-axis) compared with the transient deviation parameters as calculated from equations in terms of combination input variables (x-axis). (*a*–*c*) Function of ${\tilde{R}}_{M}$ and Le, (*d*–*f* ) function of ${\tilde{R}}_{E}$, (*g*–*i*) function of δ.

The correlations between the three predictors (${\tilde{R}}_{M}$, ${\tilde{R}}_{E}$, δ) and the outputs ($\epsilon_{\text{start}}$, $\epsilon_{\text{end}}$, $\epsilon_{\text{net}}$) of the fully transient model are captured in the equations shown on the plots of figure 11. Figure 12 shows the general accuracy of the proposed equation’s ability to predict the result of the fully transient model. The data points shown in figure 12 are the same as in figure 11 but figure 12 shows more clearly the scattering from the proposed equations. The 95% confidence values indicate the given equation’s predictive capability, where smaller values indicate greater accuracy. For example, the equation shown in figure 12*a* predicts $\epsilon_{\text{start}}$ to within ±28% for 95% of the cases considered. Reasons for the scattering can clearly be seen and understood from figure 9. For example, figure 9*a* shows the variance in $\epsilon_{\text{start}}$ due to changes to ε is not adequately captured by changes to ${\tilde{R}}_{E}$. If the input domain was made narrower (i.e. by reducing the bounds in table 3) then less scattering would be observed.

From the scatter shown in figure 12, it was concluded that $\epsilon_{\text{net}}$ can be predicted by all proposed equations with good accuracy. However, the shape of the droplet size history curve (informed by $\epsilon_{\text{start}}$ and $\epsilon_{\text{end}}$) is more challenging to predict. The parameter ${\tilde{R}}_{M}$ (combined with Le) was found to be the best predictor of these parameters, followed by ${\tilde{R}}_{E}$ and then δ. In particular, δ alone is a poor predictor of $\epsilon_{\text{end}}$. It is logical that δ would have the least predictive power since it is closely related to the leading order approximation of ${\tilde{R}}_{M}$ and ${\tilde{R}}_{E}$. The higher order effects captured by the ${\tilde{R}}_{M}$ and ${\tilde{R}}_{E}$ increase their predictive capability as they approach unity. However, δ is far simpler to compute since evaluating the integrals in equations (4.10) and (4.12) to accurately quantify ${\tilde{R}}_{M}$ and ${\tilde{R}}_{E}$ requires numerical integration except in some special cases. It may therefore seem erroneous that δ is the most accurate predictor of $\epsilon_{\text{net}}$. However, the confidence bounds are skewed significantly by the large amount of data with large ${\tilde{R}}_{M}$ and ${\tilde{R}}_{E}$ values, where $\epsilon_{\text{net}}$ is small and so large percentage errors can occur with the proposed equations. This could be corrected by adding additional terms to the $\epsilon_{\text{net}}=f({\tilde{R}}_{M},\text{Le})$ and $\epsilon_{\text{net}}=f({\tilde{R}}_{E})$ equations. However, there is little to gain from this since δ is an adequate predictor in this range (for small δ) and is simpler to evaluate.

Each ϵ parameter, therefore, has three possible predictive equations. The choice of selecting which is most effective was done on the basis of which gives the lowest 95% confidence values. It is therefore recommended that the following equations should be used to predict the transient deviation parameters

4.15 $$\begin{array}{rl} & \epsilon_{\text{start}}=\frac{-1.236}{\sqrt{\text{Le}}({\tilde{R}}_{M}-1)+1.822},\;\epsilon_{\text{end}}=\frac{0.4282}{\sqrt{\text{Le}}({\tilde{R}}_{M}-1)+0.3443}\\ \text{and}\; & \epsilon_{\text{net}}=0.779\left(\frac{1}{1-0.706\delta }-1\right). \end{array}\}$$

The validity of the QS assumption decreases as the magnitude of $\epsilon_{\text{net}}$ increases. If the magnitude of $\epsilon_{\text{net}}$, evaluated through equation (4.15), is large (e.g. $|\epsilon_{\text{net}}|>0.05$, meaning greater than 5% error), then the QS assumption is invalid. The precise value of $\epsilon_{\text{net}}$ at which the QS assumption becomes invalid is dependent on the error tolerance of the given application, but equation (4.15) provides the general method to calculate the overall percentage error from the QS assumption.

### (e) Application to real fluids

The fully transient evaporation problem has been solved for a very wide envelope of governing dimensionless numbers that encompasses many scenarios that may be encountered in practice. This section now considers real fluids to quantify the actual input variables that may be encountered and consequently quantify the error in adopting the classical QS gas phase assumption. As has been demonstrated, the difference between the fully transient and QS models is a function of 10 dimensionless numbers, four of which are dominant ($\bar{\rho }$, T, Ja, ${\bar{c}}_{p}$). However, the problem can be reduced to just three numbers as follows, at the expense of some accuracy. For T≫1, then $B_{T}\to \text{Ja}(T-1)$ and equation (4.7) can therefore be written as

4.16 $$\delta =-{\left\{\frac{8}{\pi }\left(\frac{\bar{\rho }T}{{\bar{c}}_{p}}\right)\frac{\ln[\text{Ja}(T-1)+1]}{T}\right\}}^{1/2},$$

and equation (4.16) can evaluate $\epsilon_{\text{net}}$ through equation (4.15), meaning that $\epsilon_{\text{net}}=f(T,\bar{\rho }T/{\bar{c}}_{p},\text{Ja})$. In terms of accuracy, for T=3 this approximation can lead to an ∼5% error in evaluating $B_{T}$ (where the error is always an underprediction) and equation (4.15) for $\epsilon_{\text{net}}$ has an associated uncertainty of approximately 21%. For a given ambient gas and evaporating liquid, these dimensionless numbers are a function of system temperature and pressure only ($T_{\infty }$, $P_{\infty }$). The effect of increasing $T_{\infty }$ is twofold: to increase T and decrease $\bar{\rho }$. It is for this reason that the term $\bar{\rho }T/{\bar{c}}_{p}$ is used since this is mainly a function of pressure (${\bar{c}}_{p}$ is a weak function of temperature). Similarly, Ja is mainly a function of pressure.

**Figure 13.**
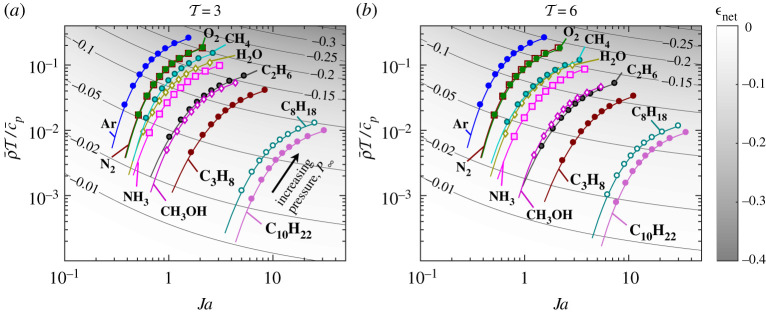
(*a*,*b*) Map of $\epsilon_{\text{net}}$ as approximated by equations (4.15) and (4.16) as a function of Ja and $\bar{\rho }T/{\bar{c}}_{p}$ for two different excess temperature ratios (T). Conditions for various substances evaporating in air at various pressures are shown. The lines begin at reduced pressure $P_{\infty }/P_{c}=0.02$ and markers are placed at increments of 0.1 from $P_{\infty }/P_{c}=0.1$ to 0.9. All fluid properties are from NIST [40], $\rho_{l}$ and L are evaluated at saturation conditions, specific heats $c_{p,\infty }$ and $c_{p}^{A}$ are evaluated at the film temperature $T_{f}=T_{\text{BP}}+(T_{\infty }-T_{\text{BP}})/3$. (Online version in colour.)

Before proceeding, the validity of the assumptions should be addressed. The key results of this study considered ideal fluids that have no critical point. However, when considering real fluids at elevated pressures, deviations from ideal behaviour can occur as the critical point is approached. The following results presented in figure 13 go up to a reduced pressure ($P_{\infty }/P_{c}$, where $P_{c}$ is the critical pressure) of 0.9, at which deviations from ideal gas behaviour will occur. The greatest non-ideal gas effects will occur near the droplet surface where temperatures are low. Addressing accurately the impact of non-ideal behaviour is beyond the scope of the present work so results are presented under the knowledge that non-ideal gas effects will lead to some errors. However, it is noted that the ideal gas equation generally underpredicts the density for a given pressure and temperature (when the compressibility factor is less that unity). Therefore, non-ideal behaviour will lead to increased gas density in the vicinity of the droplet compared with an ideal gas. A high-density gas phase (increased $\bar{\rho }$) has been shown to increase the gas phase transient effects and, therefore, it is logical that a non-ideal gas would increase the difference between the QS and fully transient models. This suggests that the results shown in figure 13 are likely to be underpredictions of the magnitude of $\epsilon_{\text{net}}$. However, this is yet to be demonstrated and is the topic of future work.

Figure 13 shows how $\epsilon_{\text{net}}$ varies in terms of the three dimensionless numbers (T, $\bar{\rho }T/{\bar{c}}_{p}$, Ja). It emerges that $\epsilon_{\text{net}}$ is overall a weak function of ambient temperature, where sensitivity to T depends on Ja. For small Ja, there is little difference moving from T=3 to 6. However, for large Ja, $\epsilon_{\text{net}}$ is marginally lower for elevated T. Inspection of equation (4.16) shows there is a T that gives a maximum magnitude of $\epsilon_{\text{net}}$ depending on Ja. For example, if Ja=1, maximum magnitude of $\epsilon_{\text{net}}$ is predicted to occur at T=2.6.

For given fluids, the dominant factor that determines the significance of gas phase transients is the system pressure. Figure 13 shows the path followed by various fluids in air as the pressure is varied from reduced pressure $P_{\infty }/P_{c}=0.02$ to 0.9. As the pressure increases, $\epsilon_{\text{net}}$ increases in all cases. Physically, this is because as pressure increases, the density of the gas surrounding the droplet increases. Hence, the thermal and mass inertia of the surrounding gas increases, meaning that more energy must be removed from the atmosphere and more vapour must be added to establish the surrounding temperature and mass fraction fields, respectively. There is a compounding effect because increasing pressure decreases the latent heat of vaporization, meaning that the droplet has a reduced amount of energy it can absorb from the atmosphere before being completely vaporized. The preceding logic is quantified by the variable ${\tilde{R}}_{E}$ (closely related to δ) which effectively compares the energy to completely vaporize the droplet with the energy to establish the QS temperature profile.

Identifying fluids that experience greater gas phase transient effects (i.e. increased $\epsilon_{\text{net}}$ magnitude) is purely a question of the fluid properties. However, from the fluids considered in figure 13, some general trends can be extracted. Generally, fluids with lower boiling points are more susceptible to greater $\epsilon_{\text{net}}$ (for a given $P_{\infty }/P_{c}$). This is because, with a lower boiling point, one can simultaneously have a high T and high $\bar{\rho }$. For a fluid with a high boiling point, the high temperature environments required to attain high T lead to low gas densities. There are some exceptions to this trend; for example, Ar shows higher $\epsilon_{\text{net}}$ than N${\;}_{2}$. This is mainly due to the low heat capacity of Ar. H${\;}_{2}$O also goes against the trend due to a much larger latent heat than other fluids, but also due to a substantially greater critical pressure. Among the alkanes, the trend is robust, with heavier components displaying lower $\epsilon_{\text{net}}$.

In terms of the magnitudes of $\epsilon_{\text{net}}$ found, it was concluded that at low pressures (approximately atmospheric), and for the cases considered, gas phase transient effects are not significant since $|\epsilon_{\text{net}}|<3\text{\%}$ for all cases. This is not to say applications do not exist where high $\epsilon_{\text{net}}$ magnitude may be experienced at atmospheric pressure, for example, by varying the ambient gas. An ambient gas with high heat capacity ($c_{p,\infty }$) and high average molecular mass (hence low $R_{\infty }$) is predicted to result in greater gas transient effects. It was also found that for heavy hydrocarbons, even at elevated pressures, gas phase transient effects can still be relatively small. For example, the maximum transient deviation found for decane at a pressure equivalent to 19 bar was 9% ($\epsilon_{\text{net}}=-0.09$). Of course, whether this error is acceptable depends on the desired accuracy in the context of the application. It is also important to note that transient effects manifest in more complex ways than only changing the total lifetime prediction. The two transient regimes discussed previously have an opposite effect on the total lifetime prediction such that a reasonably small $\epsilon_{\text{net}}$ could also display a significant departure from classical, linear, $d^{2}$-law behaviour. For reference, case (3) shown in figure 7 departs significantly from linear behaviour for a resulting $\epsilon_{\text{net}}=-0.26$.

It was found for low boiling point substances such as some of the cryogens listed, the QS assumption could lead to substantial errors at elevated pressures even at room temperature, e.g. for nitrogen evaporating under ambient conditions of 11 bar/ 315 K, $\epsilon_{\text{net}}=-0.1$. The magnitude of $\epsilon_{\text{net}}$ increases to −0.2 at a pressure of 30 bar. It is also important to note that for these substances, the initial condition of ${\tilde{\theta }}_{l,i}=-1$ is more applicable since cryogens are often stored under saturation conditions, i.e. there are negligible liquid phase transients due to droplet heating or cooling. Overall, quantifying the significance of gas phase transients is case specific for the application under consideration. The presented study provides the tools for researchers to quantify errors resulting from the QS assumption for a general application.

## 5. Conclusion

We analysed the effect of gas phase transients on the droplet evaporation process by means of an exhaustive, fully transient model with carefully controlled and clearly stated assumptions. The main output of this study is a predictive tool, equation (4.15), that can accurately predict the error caused by assuming a QS gas phase. It is proposed that this predictive tool can be applied by researchers to justify the use of the widely used QS assumption or alternatively to highlight situations where QS assumption may not be appropriate. The predictive equations provided in the main text were found to be robust over a wide range of conditions. The classical justifications of the QS assumption, i.e. $\rho_{\infty }/\rho_{l}\ll 1$ and $\rho_{\infty }/(\text{Le}\rho_{l})\ll 1$, were found to be inaccurate since an illustrative example demonstrated a 9% error in the case where $\rho_{\infty }/\rho_{l}=\rho_{\infty }/(\text{Le}\rho_{l})=0.02$ (table 2). While the quantities $\rho_{\infty }/\rho_{l}$ and $\rho_{\infty }/(\text{Le}\rho_{l})$ were found to be important parameters, these parameters alone lack significant predictive capability.

It was found that the QS assumption breaks down when the amount of energy required to establish the QS temperature field in the vicinity of the droplet is of the same order as the amount of energy required to bring the droplet into equilibrium with its environment. Also significant, and closely related, is the ratio of vapour mass accumulated in the QS concentration field compared with the mass of vapour contained within the droplet.

When the fully transient and the QS solution differ, it was found that two transient regimes exist. When a droplet is first exposed to an undisturbed atmosphere, the evaporation rate is initially faster than predicted by the QS model since the temperature and concentration profiles take time to establish around the droplet. The second transient regime occurs towards the end of droplet life and, to the present authors’ knowledge, this has not previously been explained or quantified. Towards the end of droplet life, the surface temperature gradient and concentration gradient diverge from the QS value as the droplet becomes insulated by a cold region of its own vapour, causing a reduced evaporation rate. The solution, therefore, becomes history dependent. It was found that the occurrence of this second regime is only predicted by correctly accounting for the effects of the moving droplet surface.

For a given system of fluids, it was found that gas phase transients are relatively insensitive to the ambient temperature but are highly sensitive to ambient pressure. Gas phase transients become more significant at elevated pressures primarily due to a denser gas phase with greater thermal and mass inertia. Considering a wide range of liquids evaporating in air, we show that deviation from the $d^{2}$-law can reach approximately 20% while still at subcritical pressures (e.g. liquid nitrogen in air at 30 bar). Deviations in excess of 45% were demonstrated for certain combinations of dimensionless numbers that do not necessarily correspond to real fluids. Physically, a 45% deviation means the QS assumption overpredicts droplet lifetime by a factor of approximately 1.8 compared with the fully transient solution.

The new fully transient model presented here was shown to precisely recover analytical solutions where available (e.g. heated sphere problem) and trivial cases of QS evaporation, when the ambient temperature is less than boiling, for example (T<1). Further validation was provided through recovering past results. The dimensionless approach and the assumptions made distil the problem into 10 governing dimensionless numbers, four of which have a large effect on the result. The assumptions such as constant specific heats were made to obtain the desired generality. Relaxing assumptions such as these will obtain more accurate, yet case-specific solutions.

## Data accessibility

The code to reproduce the results of this article is included in the electronic supplementary material.

## Appendix A. List of assumptions

Table 4 lists the assumptions relevant to droplet evaporation problems. Assumptions 1–5 define the class of problem considered in the present article; that of a single, spherical, pure droplet in a stagnant, infinite, isotropic gaseous medium. Assumptions 7–9 are important considerations for small droplets. Assumptions 1–22 are the full list of assumptions used in the present work and leads to the stated conclusions. Adopting all 27 assumptions (with the exception of assumption 26 if constant Γ is used) obtains Maxwell’s equation (1.1). Relaxing assumption 27, and 26 if constant mass diffusivity Γ is used, obtains the full QS solution (1.2). Further relaxing 25 obtains the infinite conductivity model. Further relaxing 24 obtains the finite or effective conductivity model. Further relaxing 23 obtains the present model.

**Table 4.** List of possible assumptions relevant to droplet evaporation problems.

no.	assumption	no.	assumption
1.	spherical droplet	15.	inviscid fluids
2.	infinite domain	16.	incompressible gas phase
3.	isolated droplet	17.	negligible thermal expansion of
4.	pure (mono-component) droplet		the liquid phase
5.	stationary droplet	18.	radiative heat transfer is neglected
6.	microgravity	19.	ideal gas
7.	the fluid is a continuum	20.	pure conduction within the liquid (no convection)
8.	surface tension effects are negligible	21.	constant gas specific heats ($c_{p}$)
9.	infinitely thin phase transition zone/	22.	constant transport properties (Γ,λ,μ)
	negligible Knudsen layer	23.	quasi-steady gas phase
10.	local phase equilibrium at the	24.	uniform liquid temperature
	droplet surface	25.	steady (constant in time)
11.	ambient gas is insoluble within droplet		liquid temperature
12.	Soret/Dufour effect neglected	26.	constant density/isothermal gas phase
13.	incompressible liquid phase	27.	no net gas velocity (Stefan velocity)
14.	constant liquid phase properties ($c_{l},\lambda_{l}$)		

## References

[1] Maxwell JC. 1877 Diffusion. In *Encyclopaedia britannica*, 9th edn, pp. 214–221. Edinburgh, Adam and Charles Black.

[2] Faeth GM. 1977 Current status of droplet and liquid combustion. Prog. Energy Combust. Sci. **3**, 191-224. (10.1016/0360-1285(77)90012-0)

[3] Law CK. 1982 Recent advances in droplet vaporization and combustion. Prog. Energy Combust. Sci. **8**, 171-201. (10.1016/0360-1285(82)90011-9)

[4] Sazhin SS. 2006 Advanced models of fuel droplet heating and evaporation. Prog. Energy Combust. Sci. **32**, 162-214. (10.1016/j.pecs.2005.11.001)

[5] Aggarwal SK, Peng F. 1995 A review of droplet dynamics and vaporization modeling for engineering calculations. J. Eng. Gas Turbines Power **117**, 453. (10.1115/1.2814117)

[6] Sazhin SS. 2017 Modelling of fuel droplet heating and evaporation: recent results and unsolved problems. Fuel **196**, 69-101. (10.1016/j.fuel.2017.01.048)

[7] Zubkov VS, Cossali GE, Tonini S, Rybdylova O, Crua C, Heikal M, Sazhin SS. 2017 Mathematical modelling of heating and evaporation of a spheroidal droplet. Int. J. Heat Mass Transfer **108**, 2181-2190. (10.1016/j.ijheatmasstransfer.2016.12.074)

[8] Yin C. 2016 Transient heating and evaporation of moving mono-component liquid fuel droplets. Appl. Therm. Eng. **104**, 497-503. (10.1016/j.applthermaleng.2016.05.098)

[9] Abramzon B, Sirignano WA. 1989 Droplet vaporization model for spray combustion calculations. Int. J. Heat Mass Transfer **32**, 1605-1618. (10.1016/0017-9310(89)90043-4)

[10] Barata J. 2008 Modelling of biofuel droplets dispersion and evaporation. Renewable Energy **33**, 769-779. (10.1016/j.renene.2007.04.019)

[11] Ray S, Raghavan V. 2020 Numerical study of evaporation characteristics of biodiesel droplets of Indian origin. Fuel **271**, 117637. (10.1016/j.fuel.2020.117637)

[12] Maqua C, Castanet G, Lemoine F. 2008 Bicomponent droplets evaporation: temperature measurements and modelling. Fuel **87**, 2932-2942. (10.1016/j.fuel.2008.04.021)

[13] Arabkhalaj A, Azimi A, Ghassemi H, Shahsavan Markadeh R. 2017 A fully transient approach on evaporation of multi-component droplets. Appl. Therm. Eng. **125**, 584-595. (10.1016/j.applthermaleng.2017.07.054)

[14] Ebrahimian V, Habchi C. 2011 Towards a predictive evaporation model for multi-component hydrocarbon droplets at all pressure conditions. Int. J. Heat Mass Transfer **54**, 3552-3565. (10.1016/j.ijheatmasstransfer.2011.03.031)

[15] Castanet G, Perrin L, Caballina O, Lemoine F. 2016 Evaporation of closely-spaced interacting droplets arranged in a single row. Int. J. Heat Mass Transfer **93**, 788-802. (10.1016/j.ijheatmasstransfer.2015.09.064)

[16] De Rivas A, Villermaux E. 2016 Dense spray evaporation as a mixing process. Phys. Rev. Fluids **1**, 1-15. (10.1103/PhysRevFluids.1.014201)

[17] Bellan J, Cuffel R. 1983 A theory of nondilute spray evaporation based upon multiple drop interactions. Combust. Flame **51**, 55-67. (10.1016/0010-2180(83)90083-4)

[18] Langmuir I. 1918 The evaporation of small spheres. Phys. Rev. **12**, 368-370. (10.1103/PhysRev.12.368)

[19] Fuchs N. 1934 Concerning the velocity of evaporation of small droplets in a gas temperature. Physikalische Zeitschrift der Sowjetunion **6**, 224-243.

[20] Spalding DB. 1979 Combustion and mass transfer. Oxford, UK: Pergamon Press.

[21] Tonini S, Cossali GE. 2012 An analytical model of liquid drop evaporation in gaseous environment. Int. J. Therm. Sci. **57**, 45-53. (10.1016/j.ijthermalsci.2012.01.017)

[22] Sobac B, Talbot P, Haut B, Rednikov A, Colinet P. 2015 A comprehensive analysis of the evaporation of a liquid spherical drop. J. Colloid Interface Sci. **438**, 306-317. (10.1016/j.jcis.2014.09.036) 25454455

[23] Bradley RS, Evans MG, Whytlaw-gray RW. 1946 The rate of evaporation of droplets; evaporation and diffusion coefficients, and vapour pressures of dibutyl phthalate and butyl stearate. Proc. R. Soc. Lond. A **186**, 368-390. (10.1098/rspa.1946.0050) 20998740

[24] Hołyst R, Litniewski M, Jakubczyk D, Kolwas K, Kolwas M, Kowalski K, Migacz S, Palesa S, Zientara M. 2013 Evaporation of freely suspended single droplets: experimental, theoretical and computational simulations. Rep. Prog. Phys. **76**, 034601. (10.1088/0034-4885/76/3/034601) 23439452

[25] Rana AS, Lockerby DA, Sprittles JE. 2019 Lifetime of a nanodroplet: kinetic effects and regime transitions. Phys. Rev. Lett. **123**, 154501. (10.1103/PhysRevLett.123.154501) 31702290

[26] Millán-Merino A, Fernández-Tarrazo E, Sánchez-Sanz M. 2021 Theoretical and numerical analysis of the evaporation of mono- and multicomponent single fuel droplets. J. Fluid Mech. **910**, A11. (10.1017/jfm.2020.950)

[27] Aggarwal SK, Tong AY, Sirignano WA. 1984 A comparison of vaporization models in spray calculations. AIAA J. **22**, 1448-1457. (10.2514/3.8802)

[28] Sirignano WA. 2010 Fluid dynamics and transport of droplets and sprays, 2nd edn. Cambridge, UK: Cambridge University Press.

[29] Talbot P, Sobac B, Rednikov A, Colinet P, Haut B. 2016 Thermal transients during the evaporation of a spherical liquid drop. Int. J. Heat Mass Transfer **97**, 803-817. (10.1016/j.ijheatmasstransfer.2015.12.075)

[30] Hubbard GL, Denny VE, Mills AF. 1975 Droplet evaporation: effects of transients and variable properties. Int. J. Heat Mass Transfer **18**, 1003-1008. (10.1016/0017-9310(75)90217-3)

[31] Zhu GS, Reitz RD, Aggarwal SK. 2001 Gas-phase unsteadiness and its influence on droplet vaporization in sub- and super-critical environments. Int. J. Heat Mass Transfer **44**, 3081-3093. (10.1016/S0017-9310(00)00349-5)

[32] Azimi A, Arabkhalaj A, Ghassemi H, Shahsavan Markadeh R. 2017 Effect of unsteadiness on droplet evaporation. Int. J. Therm. Sci. **120**, 354-365. (10.1016/j.ijthermalsci.2017.06.023)

[33] Tonini S, Cossali GE. 2018 Modeling of liquid drop heating and evaporation: the effect of drop shrinking. Comput. Therm. Sci. Int. J. **10**, 273-283. (10.1615/ComputThermalScien.2018021330)

[34] Matalon M, Law CK. 1983 Gas-phase transient diffusion in droplet vaporization and combustion. Combust. Flame **50**, 219-229. (10.1016/0010-2180(83)90063-9)

[35] Bird RB, Stewart WE, Lightfoot EN. 2001 Transport phenomena, 2nd edn. New York, NY: John Wiley and Sons Inc.

[36] Cossali GE, Tonini S. 2019 An analytical model of heat and mass transfer from liquid drops with temperature dependence of gas thermo-physical properties. Int. J. Heat Mass Transfer **138**, 1166-1177. (10.1016/j.ijheatmasstransfer.2019.04.066)

[37] Ha VM, Lai CL. 2001 The onset of stationary Marangoni instability of an evaporating droplet. Proc. R. Soc. Lond. A **457**, 885-909. (10.1098/rspa.2000.0697)

[38] Sazhin S. 2014 Droplets and sprays. Berlin, Germany: Springer.

[39] Tonini S, Cossali GE. 2016 Moving boundary and time-dependent effects on mass transfer from a spherical droplet evaporating in gaseous environment. In *ICMF-2016 – 9th Int. Conf. on Multiphase Flow, Firenze, Italy*.

[40] NIST Chemistry WebBook, Thermophysical Properties of Fluid Systems. See https://webbook.nist.gov/chemistry/fluid/.

